# RadicalPy: A Tool for Spin Dynamics Simulations

**DOI:** 10.1021/acs.jctc.4c00887

**Published:** 2024-10-29

**Authors:** Lewis M. Antill, Emil Vatai

**Affiliations:** † Department of Chemistry, University of Oxford, Physical and Theoretical Chemistry Laboratory, South Parks Road, Oxford OX1 3QZ, U.K.; ‡ High Performance Artificial Intelligence Systems Research Team, RIKEN Center for Computational Science, 7 Chome-1-26 Minatojima Minamimachi, Kobe, Hyogo 650-0047, Japan

## Abstract

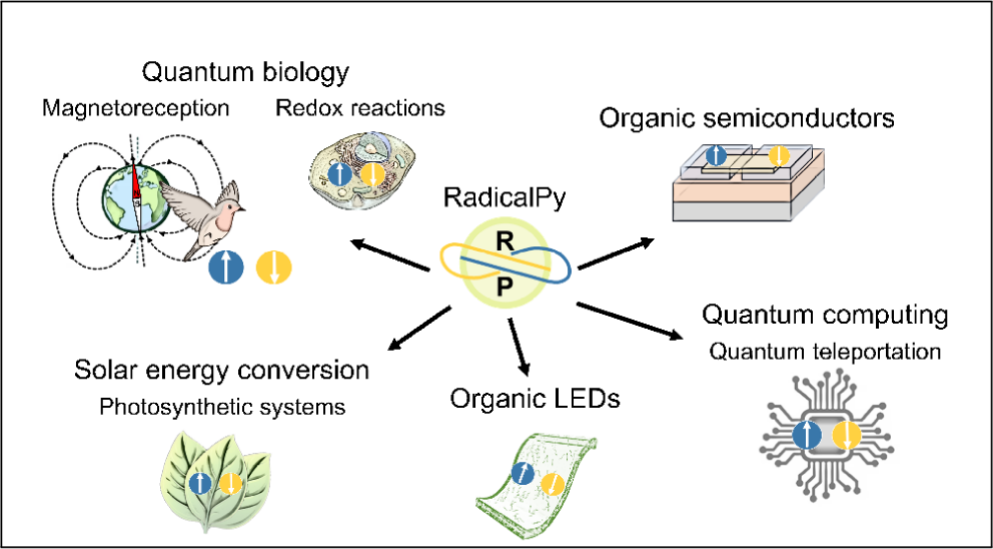

Radical pairs (electron–hole pairs, polaron pairs) are transient reaction intermediates that are found and exploited in all areas of science, from the hard realm of physics in the form of organic semiconductors, spintronics, quantum computing, and solar cells to the soft domain of chemistry and biology under the guise of chemical reactions in solution, biomimetic systems, and quantum biology. Quantitative analysis of radical pair phenomena has historically been successful by a few select groups. With this in mind, we present an intuitive open-source framework in the Python programming language that provides classical, semiclassical, and quantum simulation methodologies. A radical pair kinetic rate equation solver, Monte Carlo-based spin dephasing rate estimations, and molecule database functionalities are implemented. We introduce the *kine-quantum* method, a new approach that amalgamates classical rate equations, semiclassical, and quantum techniques. This method resolves the prohibitively large memory requirement issues of quantum approaches while achieving higher accuracy, and it also offers wavelength-resolved simulations, producing time- and wavelength-resolved magnetic field effect simulations. Model examples illustrate the versatility and ease of use of the software, including the new approach applied to the magnetosensitive absorption and fluorescence of flavin adenine dinucleotide photochemistry, spin–spin interaction estimation from molecular dynamics simulations on radical pairs inside reverse micelles, radical pair anisotropy inside proteins, and triplet exciton pairs in anthracene crystals. The intuitive interface also allows this software to be used as a teaching or learning aid for those interested in the field of spin chemistry. Furthermore, the software aims to be modular and extensible, with the aim to standardize how spin dynamics simulations are performed.

## 1 Introduction

The interaction of a pair of radicals, known as a radical pair (RP), was first observed as anomalous line intensities in electron paramagnetic resonance (EPR) spectroscopy by Fessenden and Schuler in 1963^1^ and in 1967 by Bargon, Fischer, and Johnsen^2^ and independently Ward and Lawler^3^ observed similar anomalies in nuclear magnetic resonance (NMR) spectroscopy. These phenomena are known as chemically induced dynamic electron polarization (CIDEP) and chemically induced dynamic nuclear polarization (CIDNP), respectively. In 1969, a new theory was postulated to explain these anomalous intensities by Closs^4^ and independently by Kaptein and Oosterhoff,^5^ which is known as the radical pair mechanism (RPM).

Since the 1970s, there have been considerable advances in the theoretical framework and many methods and experiments have been developed, creating the field of Spin Chemistry.^6^ This field extends from chemical systems encompassing the solid and liquid state, to both biological systems and material science, with the application of magnetic field strengths ranging from very weak fields of tens of micro-Tesla up to tens of Tesla. Important applications in areas of science include magnetoconductivity and magnetoelectroluminescence in organic semiconductors,^7,8^ qubits in quantum computing,^9^ enhancement of NMR sensitivity,^10,11^ and photosynthetic systems for solar energy conversion.^12^ We are motivated to address the spin dynamics simulations required for these problems by introducing *RadicalPy*. Moreover, *RadicalPy* can also be used for spintronics simulations, since the spintronics community has recently reinvented the nomenclature developed for the techniques and theory created by spin chemists.^13^

In recent times, the radical pair mechanism has gained popularity with nonspecialists through its proposed involvement in biological magnetoreception^14^ and quantum biology.^15^ Furthermore, the lack of reproducibility in the effects of a magnetic field on radical pairs in biological reactions^16−20^ calls for a standardized method to simulate experimental results. We aim ambitiously that *RadicalPy* will be a standard for the community to use and develop.

Current toolboxes, such as *EasySpin*(21,22) and *Spinach*(23) focus on magnetic resonance techniques, NMR and EPR, where spin dynamics simulations offer little or no designated functionality for radical pair specific problems and require proprietary commercial software.^24^*MolSpin* software,^25,26^ the recent and more versatile software in terms of radical pair simulations is an open-source alternative, implemented in the C++ programming language and uses a custom scripting language. This approach is necessary for larger simulations; however, because of the significantly higher barrier of entry, it is not adequate for small- and medium-sized simulations and rapid prototyping.

With this in mind, our aim is to democratize spin dynamics simulations for the experimentalist, by developing an intuitive, object-oriented, open-source^27^ framework in the Python programming language. We hope that this versatile framework provides the means for students and researchers to perform correct and complex radical pair dynamics simulations with relative ease, making it ideal as a teaching or learning tool for creating quick simulations on the fly.

## 2 Methods

*RadicalPy* includes classical, semiclassical, and quantum simulation methods. The toolbox offers an isotope and molecule database (which includes spin multiplicities, magnetogyric ratios, and hyperfine coupling constants), allowing the solution of the Liouville–von Neumann equation (LvN) to be solved in both Hilbert and Liouville space with relative ease. This unique aspect removes the danger of common errors, such as errors with unit conversions, and also alleviates the issue of not having access to or the ability to perform density functional theory (DFT) calculations. Currently, DFT files are input manually; however, parsers for the popular DFT calculation software *Gaussian*(28) and *ORCA*(29) will appear in a future version of *RadicalPy*, automating this process. General kinetics and relaxation superoperators, such as Haberkorn,^30^ Jones–Hore,^31^ singlet–triplet dephasing (STD),^32^ and random fields relaxation (RFR),^33^ are easily incorporated. Furthermore, the Schulten–Wolynes semiclassical approach allows the user to remove the computational burden of large multinuclear spin systems.^34^

One of the unique features of *RadicalPy* are the classical simulations, such as Monte Carlo random walk methodologies^35,36^ for modeling molecular diffusion of free or encapsulated RPs (e.g., micelles or vesicles). The results of which, as well as the ability to import molecular dynamics (MD) trajectories, can be used to calculate the time evolution of the exchange and dipolar couplings, and estimate spin dephasing rates.^33^ Pulsed and continuous-wave absorption and fluorescence experiments can be simulated with kinetic rate equations based on the relaxation mechanism (RM).^37^ Finally, a new approach named kine-quantum allows the user to input complex photochemical reactions, in the form of kinetic rate equations, to be integrated into semiclassical/quantum Hamiltonians in Liouville space. The advantage of this approach is that spin–spin interactions and relaxation superoperators can be incorporated with a complete photocycle containing experimentally determined rate constants, such as excitation, fluorescence decay, and intersystem crossing rate constants. Furthermore, as we can simultaneously monitor the ground state and all excited state populations, one can estimate the spectral time evolution and magnetic field dependence by incorporating reference spectra. This becomes important in special cases like flavin adenine dinucleotide (FAD), where the radical pair is in equilibrium with the triplet excited state and consequently exhibits magnetic field effects (MFEs). The kine-quantum method also allows the user to acquire the time evolution of the *B*_1/2_. All of the above can be achieved in one simulation, as demonstrated in Figure 4, providing a more accurate description of the system under investigation and greatly reducing computational costs. Classical, semiclassical, and quantum methodologies are described in the Supporting Information.

### 2.1 New Simulation Approach—Kine-Quantum

The motivation behind the development of our new simulation method was due to current limitations in the ability to perform accurate simulations of experimental results. Current constraints include memory requirements for quantum simulations. An example of 15 nuclei have been successfully simulated with a large computer cluster,^38^ however, these simulations do not include all nuclei of the radical pair (27 nuclei for the FAD-Trp radical pair) and are currently not feasible. The reason for this limitation is that in Hilbert space, the memory requirements are , while in Liouville space it is , where *N* represents the number of spins (see Table S1). Methods have been developed to reduce the size of the spin Hamiltonian,^39^ however, a computer cluster is still required to simulate a large number of spins in Liouville space. To alleviate this problem, we use the Schulten–Wolynes semiclassical methodology that has a constant memory requirement by restricting the Hamiltonian to  elements in Liouville space.

**Figure 1:**
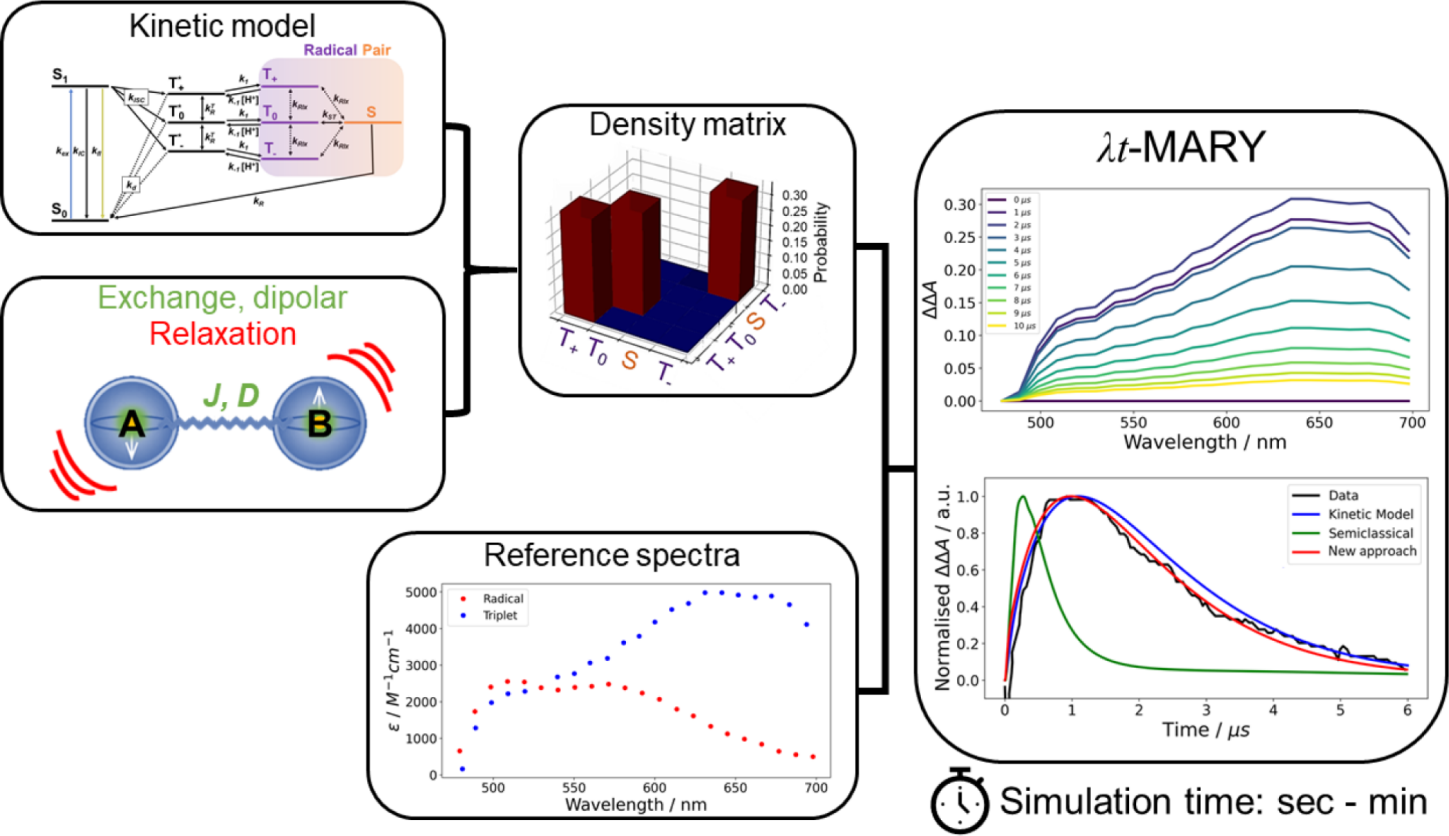
Workflow for kine-quantum. Kinetic rate equations, semiclassical, and quantum methodologies are amalgamated for the density matrix calculation. The density matrix represents the initial populations of the triplet born radical pair before spin-state mixing occurs. The density matrix is a simplified version of that used in the calculation; please refer to Figure 2 for a full description. Reference spectra are then applied to produce time- and wavelength-dependent magnetic field effect (λ*t*-MARY) simulation spectra. Parameters are found in Table S2. The script is found in the Supporting Information and is available at examples/kinetics_fad_semiclassical_wavelength.py.

The method for simulating time-resolved magnetically affected reaction yield (MARY) spectra outlined in ref. (40) utilized a semiclassical approach. However, this technique cannot capture the time dependence of the magnetic field effect on the photochemistry of FAD as simple kinetic models, i.e., Haberkorn and exponential kinetics operators, are used.^40^ This can be seen in the example in Figure 1 (lower plot, green curve), where an attempt was made to simulate the transient absorption decay kinetics of FAD with the semiclassical methodology described in ref. (40). One can see that this model has failed to capture the lifetime of the radical pair with exponential and Haberkorn kinetics operators. Typically, kinetic rate equations are used to reproduce experimental time decays with more accuracy,^37^ as shown in Figure 1 (lower plot, blue curve). The kinetic model has captured the rise and decay of the experimental data reasonably well; however, the original kinetic modeling methodology developed by Hayashi and Nagakura has limitations.^37^ Such limitations include the exclusion of full magnetic field dependence (currently limited to magnetic field off/on (at the saturation level), spin–spin interactions, and relaxation superoperators. However, recently, an approach implementing a kinetic-based model, which includes hyperfine and relaxation contributions to singlet–triplet interconversion, into quantum spin dynamics simulations (at arbitrary magnetic field strengths) has been reported.^41,42^

We have developed a new technique for incorporating chemical rate equations into quantum simulations, which allows the use of spin–spin interactions and relaxation superoperators (Figure 1). The new approach also allows reference spectra to be included in the simulation to produce time- and wavelength-resolved magnetic field effects (MFEs), which we call kine-quantum. Figure 1 shows that the new methodology (using kine_quantum_mary) reproduces the experimental data for MFEs on the photochemistry of FAD at acidic pH. The same simulation produced the MFE shown in Figure 3, which outperforms the accuracy of pure quantum MARY simulations and also gives rise to the transient absorption and fluorescence kine-quantum spectra displayed in Figure 4.

The use of kinetic models means that complex biological/chemical reactions can be produced quickly and easily with our new kinetic rate equation solver (RateEquation) and incorporated into the semiclassical/quantum simulation to produce accurate simulations of experimental data. The added bonus of this approach is that the memory requirement for these simulations is independent of the number of simulated spin electrons or nuclei and can be performed on commercial laptops (see Figure 3).

#### 2.1.1 Details of the Kine-Quantum Method

The kinetic rate equations describe the complete photochemistry of the system, e.g., the ground state, the singlet excited state, and the radical pair. However, the rate equations that describe the kinetics of the radical pair can be interpreted as a superoperator in the Liouville space of a two electron spin radical pair system.

**Figure 2:**
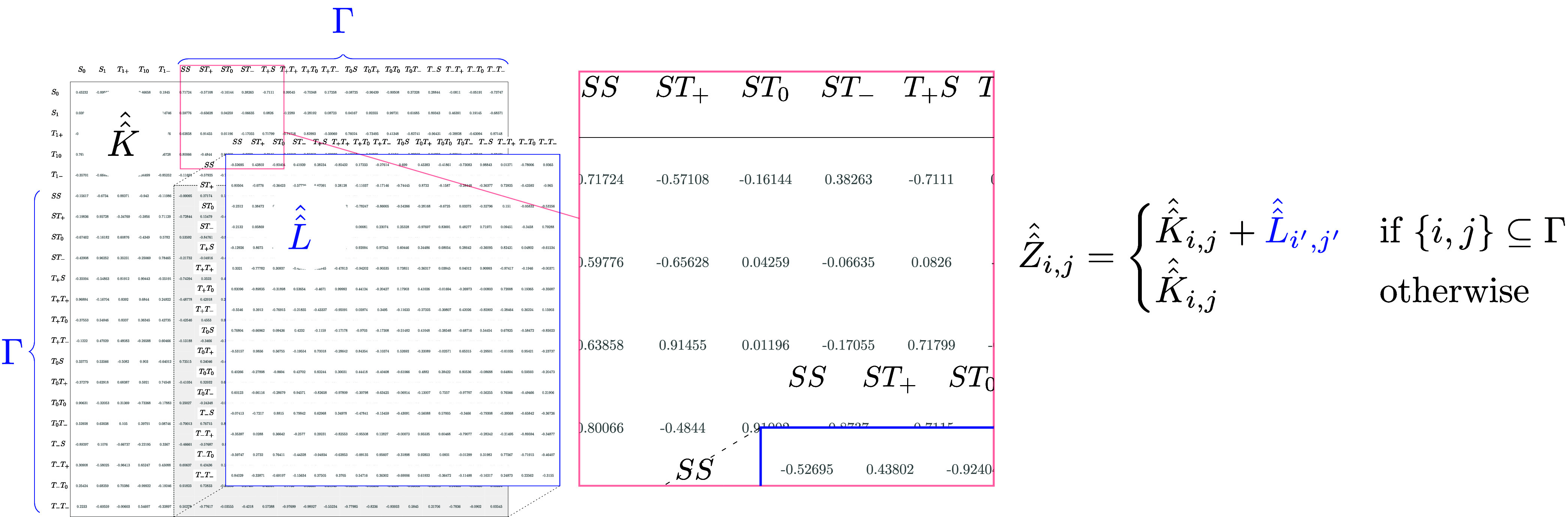
Construction of the kine-quantum superoperator, . Γ is the subset of row/column indices of  corresponding to the radical pair states. Explicitly, the 16 radical pair states encompassing Γ are *SS*, *ST*_+_, *ST*_0_, *ST*_–_, , , , , , , , , , , , and . The other states, i.e., nonradical pair states included in , are *S*_0_, *S*_1_, , , and , which gives a total number of 21 states for  in this example for FAD. The index pair,  of the Liouvillian correspond to the index pair,  of the kinetics superoperator, which share the same radical pair states.

The rate equations naturally induce a kinetics superoperator, : each row and column of the matrix correspond to a state of the system, and the rate constants that connect them are the entries in the matrix. This matrix corresponds to the adjacency matrix representation of the rate equations as a directed graph. The kinetics superoperator, , is combined with the Liouvillian, , to form the kine-quantum superoperator, , as described in Figure 2.

The Liouvillian, , consists of coherent processes and relaxation superoperators. The exchange and dipolar interactions are described by the Hamiltonians,
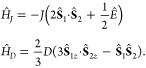
1The interactions between the electrons of the radical pair and the external magnetic field (Zeeman) and nearby nuclei (hyperfine) in the molecule(s) are defined using the Schulten–Wolynes semiclassical approach,^34^
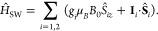
2Relaxation superoperators, , which are supported by *RadicalPy*, can also be incorporated into . The Liouvillian is therefore,
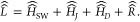
3The time dependence of the initial state, ρ_0_, evolves with the kine-quantum propagator,
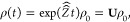
4which follows the regime of the Schulten–Wolynes semiclassical approach.^34^ Refer to the Supporting Information for a more detailed explanation of the notation.

### 2.2 Molecular Dynamics Simulations

The AOT (sodium bis(2-ethylhexyl) sulfosuccinate) water–isooctane system was constructed using the Micelle Maker online tool.^43^ Molecular dynamics simulations were performed using the NAMD package.^44,45^ The CHARMM36 force field was used for lipids and tryptophan^46,47^ and additional parametrizations for FAD.^48−50^ Periodic boundary conditions were adopted, and the Ewald summation method of the particle mesh was used to evaluate long-range Coulomb interactions. Van der Waals interactions were treated using a smooth cutoff of 12 Å with a switching distance of 10 Å. The simulation temperature was 310 K, controlled with the Langevin thermostat.^45^ A constant pressure of 1 atm for equilibrium simulations was obtained using the Langevin piston Nosé–Hoover method.^51^ The SHAKE algorithm was used to constrain bonds including hydrogen atoms at their respective equilibrium distances. After 10,000 NAMD^45^ minimization steps, equilibration simulations were performed with a solvent modeled through the TIP3P parameter set^52^ for the inner water pool, with isooctane molecules surrounding the AOT micelle. The water-to-surfactant ratio was *w*_15_, producing a micelle diameter of 60 Å. A 1:1 ratio of Na:AOT molecules was assumed to stabilize the overall charge of the system. After equilibration, temperature controlled production simulations (310 K) were performed within the statistical ensembles of NVT. All MD simulation results were analyzed with VMD.^53^

We will now discuss the advantages of kine-quantum over pure quantum methods and provide examples using the other methods provided by *RadicalPy*, which are described in the Supporting Information.

## 3 Results and Discussion

### 3.1 Comparison of Simulation Methods

**Figure 3:**
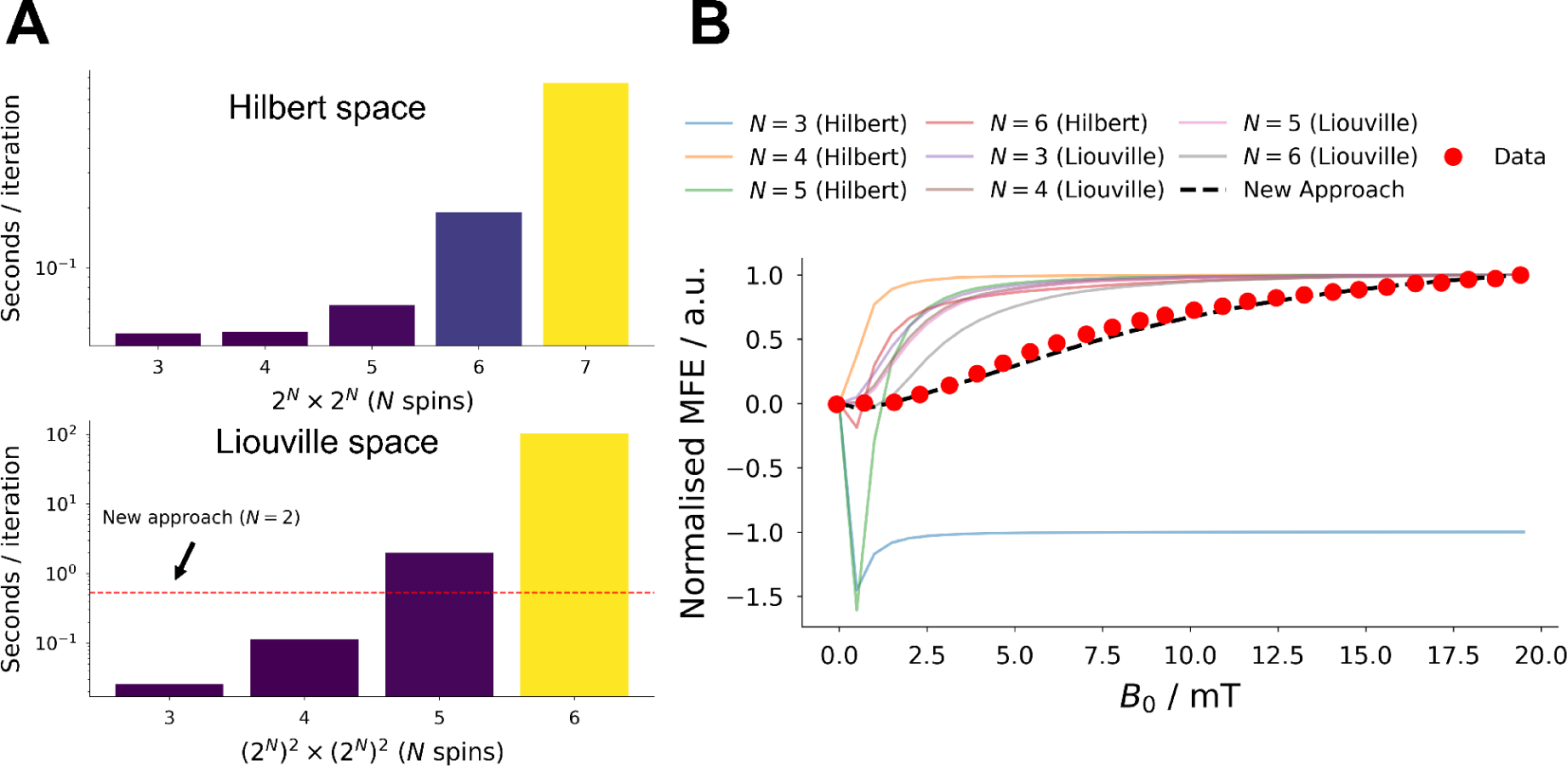
MARY simulation performance and accuracy benchmark for *RadicalPy*. (A) Performance test in both the Hilbert and Liouville spaces for an increasing number of spins. Simulations were performed until the calculation exceeded the memory capacity of a commercial laptop. The new approach is performed in Liouville space and only uses two spins. (B) Accuracy test for the above MARY simulations with increasing number of spins. The experimental result was unsuccessfully reproduced in all pure quantum simulations. The experiment was successfully modeled with the new kine-quantum approach (see the main text for details). Simulations were performed on a commercial laptop: Microsoft Surface Book 2; OS: Windows 10 Pro; CPU: Intel Core i7-8650U CPU @ 1.90 GHz 2.11 GHz; RAM: 16 GB, 1867 MHz. Storage: Samsung 512 GB M.2 SSD. Parameters are found in Table S2. The script is found in the Supporting Information and is available at examples/kinetics_fad_semiclassical_wavelength.py.

A comparison was made between quantum simulation methods and the new kine-quantum approach, on a commercial laptop, to simulate an experimental MARY spectrum for FAD at acidic pH (Figure 3). First, we look at the accuracy of the MARY spectrum simulations for the pure quantum and kine-quantum methodologies. Hilbert space simulations do not capture the shape of the experimental data and overestimate the low field effect (LFE).^54^ The Liouville space simulations (including relaxation) also fail to reproduce the experimental data. The 6-spin Liouville space quantum simulation is closest to the experimental data, but it is kine-quantum that reproduces the data exceedingly well. The performance difference between the two simulations is stark, where the 6-spin model required *ca.* 1 h (102 s/it) to complete, with the kine-quantum taking a fraction of the time 21 s (0.53 s/it). These benchmarks show that our new approach outperforms traditional quantum-based MARY simulations in both accuracy and performance.

Now, we demonstrate the versatility of *RadicalPy* with model examples. First, we simulate the photochemistry of FAD at acidic pH with the kine-quantum technique. The subsequent illustration of importing center-of-mass to center-of-mass distances acquired from a molecular dynamics simulation of an FAD^•–^–WH^•+^ radical pair encased in a sodium bis(2-ethylhexyl) sulfosuccinate (AOT) reverse micelle, which are used to estimate the exchange interaction and autocorrelation curve thereon, to estimate spin dephasing rates and the time-resolved MARY spectrum (*t*-MARY). Then a simple example of radical pair anisotropy in the proposed magnetoreceptor cryptochrome protein, with and without dipolar coupling. The final example moves away from radical pairs and into the realm of spintronics, where spin-correlated triplet exciton pairs (SCTEP) in an anthracene crystal are illustrated.

### 3.2 Radical Pairs in Solution

Flavin-based radical pairs have gained much attention due to the importance of riboflavin (vitamin B_2_), flavin mononucleotide (FMN), and flavin adenine dinucleotide (FAD) in numerous biological chemical reactions. They are present in all kingdoms of life and commonly act as electron transfer cofactors in flavoproteins.^55^ The magnetosensitivity of FAD photochemistry was first demonstrated below pH 3.6, where the flavin moiety is in an open form.^56^ The open form has a negligible exchange interaction, which allows singlet-triplet (ST) mixing to occur in the transient radical pair and to produce an MFE. It was thought that above pH 3.6, the exchange interaction is sufficiently large due to the closed-form conformation, therefore inhibiting ST-mixing and MFEs. However, with the advancement of instrument sensitivity, magnetic field effects on the photochemistry of FAD at physiological pH were revealed, where only 20–30% of the molecules are in the open form.^57^

The photochemistry of FAD is somewhat different from that of intermolecular flavin reactions because the radical pair is formed on the same molecule. This intramolecular electron transfer reaction creates a biradical between the isoallaxazine ring and the adenine moiety (Figure 1). At low pH, the radical pair is in equilibrium with the flavin triplet excited state and extends the lifetime of the radical pair.^56,57^ As pH increases, this equilibrium is gradually removed and the lifetime of the radical pair and the magnitudes of MFE and *B*_1/2_ decrease.^57^

**Figure 4:**
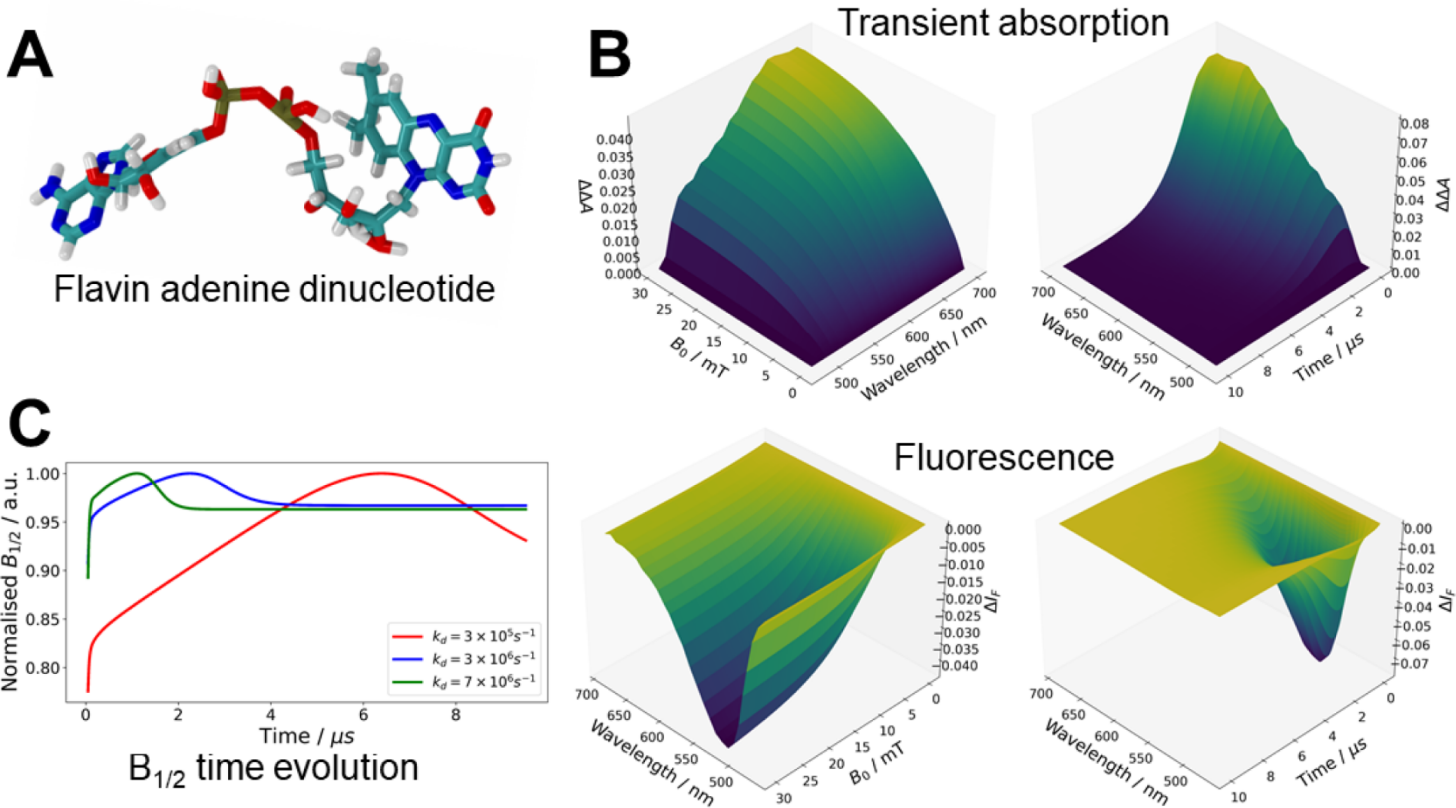
(A) The chemical structure of flavin adenine dinucleotide (FAD). (B) kine-quantum simulations of transient absorption and emission spectra for FAD. Magnetic, time, and wavelength dependencies are shown. (C) Investigation into the origin of the shape of the *B*_1/2_ time evolution for FAD. See the main text for details. The photochemical reaction scheme for FAD is shown in Figure 1. Parameters are found in Table S2. The script is found in the Supporting Information and is available at examples/kinetics_fad_semiclassical_wavelength.py.

The kine-quantum approach also produces the time evolution of the *B*_1/2_ of the radical pair under investigation. Experimental evidence has shown that the *B*_1/2_ for FAD at acidic pH gradually increases and then begins to decrease with time.^58^ Further insights will be discussed in a future publication, but we will briefly discuss a possible mechanism (reproduced with kine-quantum simulations) to explain this observation. The flavin–adenine radical pair is in equilibrium with the triplet excited state (see Figure 1 – kinetic model), which extends the lifetime of the radical pair and allows spin relaxation to broaden the MARY curve over time. However, the triplet excited state recombines to the electronic ground state of the photocycle on a longer time scale. As the triplet excited state begins to recombine to the singlet ground state, the equilibrium between the radical pair and the triplet excited state starts to disappear, shortening the lifetime of the radical pair and subsequently narrowing the MARY spectra and the *B*_1/2_. We investigated this hypothesis by increasing the triplet excited state recombination rate, *k*_*d*_, and, indeed, the point of decline in the *B*_1/2_ appears on earlier timescales (Figure 4C).

The time-resolved spectra for FAD at acidic pH are faithfully reproduced by our new method, as shown in Figures 1 and 3. The accurate simulation of the transient absorption and fluorescence spectra (Figure 4) for this biomolecule was due to the inclusion of random fields relaxation (eq S39) and singlet–triplet dephasing (eq S36). Again, showing the versatility of the new approach, we hope that this will be of use to experimentalists in the field.

This example exhibits our new approach for simulating the magnetosensitivity of the FAD biradical photochemistry (soliton–antisoliton pair) in solution. Molecular dynamics simulations include physiochemical properties such as Coulomb interactions, van der Waals interactions, temperature, pressure, and water molecules. The following example demonstrates this approach, including quantum mechanical simulations on an FAD^•–^–WH^•+^ radical pair in an AOT reverse micelle, to estimate the spin dephasing rate, which is used to simulate a *t*-MARY spectrum and time evolution of the *B*_1/2_ of the dynamics involved.

### 3.3 Radical Pairs in Micelles

The following molecular dynamics simulation and analysis were performed on a pair of FAD^•–^–WH^•+^ radicals encapsulated in an AOT reverse micelle (water and isooctane, *w*_0_ = 15) in the NAMD/VMD workspace.^44,45,53^ The flavin and tryptophan radicals are constructed using the Molecule function and the molecule database, as shown in the following code snippet,

flavin = Molecule.all_nuclei(“flavin_anion”)
trp = Molecule.all_nuclei(“tryptophan_cation”)

all_nuclei is used as this simulation utilizes the Schulten–Wolynes semiclassical approach.^34^ The radical pair is constructed for the semiclassical simulation with the SemiclassicalSimulation object, as shown in the following code snippet,

sim = SemiclassicalSimulation([flavin, trp])

**Figure 5:**
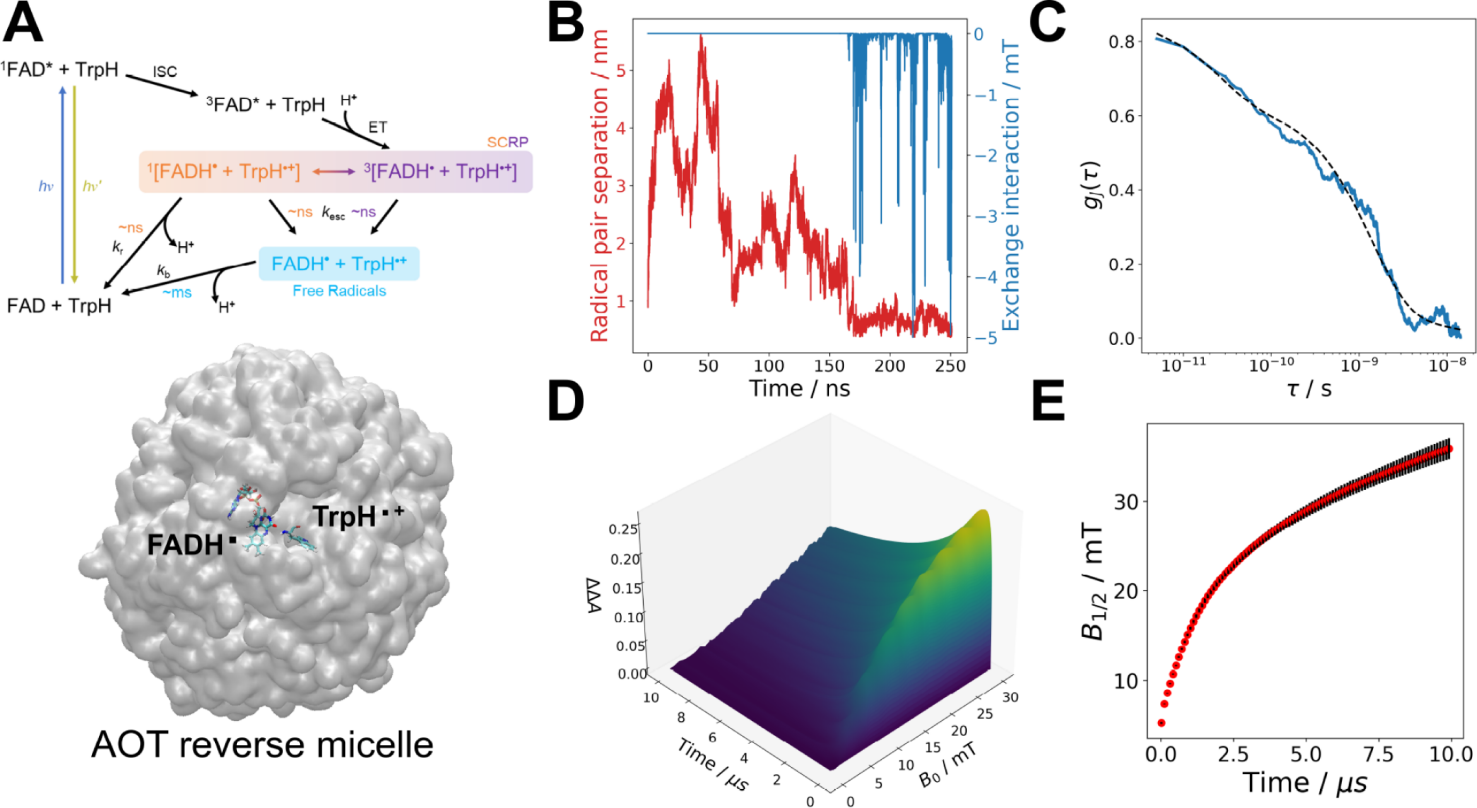
(A) A photochemical reaction scheme for FAD and Trp in solution. Below is a QuickSurf representation of the AOT reverse micelle with Licorice representations of FAD^•–^ and WH^•+^ encapsulated inside the reverse micelle. (B) Time evolution of the center-of-mass to center-of-mass radical pair separation and the exchange interaction between the two radicals. (C) Autocorrelation plot of the exchange interaction with 100 exponential fits, producing a correlation time (τ_c_) of 1.65 ns. (D) A simulated *t*-MARY spectrum for an FAD^•–^–WH^•+^ radical pair plotting the magnetic field effect () against the magnetic field (*B*_0_) and time. Singlet–triplet spin relaxation is included with a rate constant of *k*_STD_ = 1.1 × 10^7^ s^–1^, estimated from the MD simulation. (E) Time evolution of *B*_1/2_ obtained from Lorentzian fits to the *t*-MARY shown in (D). The error bars were obtained by taking the square root of the covariance matrix of the multiexponential. Parameters are found in Table S3. The script is found in the Supporting Information and is available at examples/semiclassical_micelles.py.

The center-of-mass to center-of-mass (COM–COM) distance of the FAD^•–^–WH^•+^ radical pair was taken at every time step of the MD simulation to calculate the fluctuations in the exchange interaction, *J*, over time with the estimations.exchange_interaction_in_solution_MC function. The value of J0 was adjusted (J0 = 5) to correspond to the experimentally determined value of *J* for FAD in aqueous solution.^59^ The time evolution of the COM–COM distance and *J* is shown in Figure 5B.

The MD trajectory shows that the radical pair separates at early time scales and then has numerous re-encounters as time goes on, which is mediated via π–π stacking between the Trp and FAD moieties.

The correlation time of the modulation in the exchange interaction induced by π-π stacking can be approximated by autocorrelation and exponential fitting. This can be easily achieved in *RadicalPy* with the function estimations.autocorrelation_fit, which calculates the autocorrelation and fits a multiexponential (default = 100) to the autocorrelation curve.

The correlation time for the exchange interaction, shown in Figure 5C, is 1.65 ns and is used to calculate the spin dephasing rate, *k*_STD_, with the function estimations.k_STD that produces a rate of 1.1 × 10^7^ s^–1^. This spin dephasing rate is consistent with experimental results in radical pairs in reverse micelles and is the main cause of spin relaxation in encapsulated radical pairs.^40^ The *t*-MARY was calculated with the semiclassical_mary function, as shown in the following code snippet,

results = semiclassical_mary(
  sim=sim,
  num_samples=num_samples,
  init_state=State.TRIPLET,
  ts=ts,
  Bs=Bs,
  D=0,
  J=0,
  triplet_excited_state_quenching_rate=triplet_excited_state_quenching_rate,
  free_radical_escape_rate=free_radical_escape_rate,
  kinetics=[Haberkorn(recombination_rate, State.SINGLET)],
  relaxations=[SingletTripletDephasing(kstd)],
  scale_factor=0.005,
)

Where the triplet born radical pair is defined in the function input init_state = State.TRIPLET. The kinetics of the photocycle include triplet excited state quenching, escape of the free radical, and singlet recombination. The two former kinetic processes are described by exponential models^40^ and the latter by the Haberkorn superoperator.^30^ The relaxation rate estimated by the MD simulation, as described above, is input into the singlet–triplet dephasing superoperator^32^ (all parameters and methods are described in the Supporting Information).

The *B*_1/2_ values were calculated with the utils.Bhalf_fit function to the MARY spectra in Figure 5D, and its time evolution is shown in Figure 5E. The *B*_1/2_ characterizes the sigmoidal dependence of the radical pair on the intensity of the external magnetic field.^60,61^ The *B*_1/2_ value depends on the specific characteristics of the radical pair, its lifetime, hyperfine interactions, and spin relaxation. The predicted *B*_1/2_ value using the Weller equation,^60^ which is supported by *RadicalPy* with the function estimations.Bhalf_theoretical_hyperfine, is 2.96 mT. The Weller equation estimates *B*_1/2_ using hyperfine couplings only and does not agree with the simulation values displayed in Figure 5E and experimental results in general.^61^ The reason behind the discrepancies in the predicted and simulated values is spin relaxation. This example exemplifies the importance of including spin relaxation in simulations, which is easily incorporated in *RadicalPy*, and is discussed in the Supporting Information.

The following example steps into a more complex arena with a radical pair inside a protein. Now, the molecule can no longer freely move in space, and anisotropic interactions come into play.

### 3.4 Radical Pairs in Proteins

The radical pair mechanism was first proposed as a process that could explain how migratory birds sense the magnitude and inclination of the geomagnetic field for navigation purposes.^34^ In 2000, the cryptochrome (Cry) protein was nominated as the magnetosensing biomolecule that uses radical pairs for such a purpose.^62^ Magnetic field effects have been shown to exist in purified Cry proteins from plants^63^ and animals,^64,65^ however experiments on plants and fruit flies have not been reproduced.^66,67^ Currently, experiments in birds have been proven consistent with the radical pair mechanism.^68−70^

The anisotropy of the radical pair and, therefore, the compass sensing ability have been described in the Supporting Information and will be demonstrated here in a toy model radical pair (FAD^•–^–Z^•^) and a radical pair based on the protein crystal structure of European robin cryptochrome 4a (FAD^•–^–WH^•+^, *Er*Cry4a).^71^ The FAD^•–^–Z^•^ radical pair construction is shown in the following code snippet,

flavin = Molecule.fromisotopes(isotopes=[“14N”], hfcs=[fad_n5_hfc])
Z = Molecule(“zorro”, [])
sim = HilbertSimulation([flavin, Z])

Similarly, for the FAD^•–^–WH^•+^ radical pair,

flavin = Molecule.fromisotopes(isotopes=[“14N”], hfcs=[fad_n5_hfc])
trp = Molecule.fromisotopes(isotopes=[“1H”], hfcs=[trp_hbeta_hfc])
sim = HilbertSimulation([flavin, trp])

Where the hyperfine coupling tensors are provided in the Supporting Information. The anisotropy simulations were performed with the experiments.anisotropy function, as shown in the following,

results = anisotropy(
  sim,
  init_state=State.SINGLET,
  obs_state=State.SINGLET,
  time=time,
  theta=theta,
  phi=phi,
  B0=B0,
  D=dipolar,
  J=0,
  kinetics=[kinetics.Exponential(k)],
)

**Figure 6:**
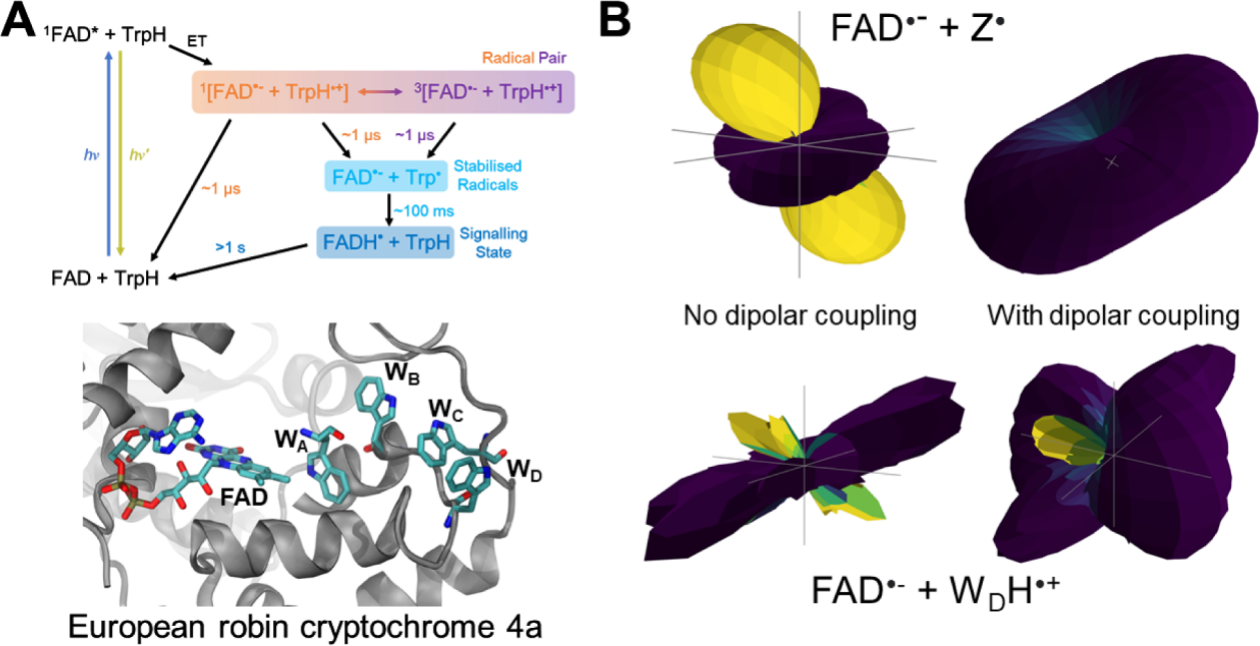
(**A**) The proposed magnetosensitive photochemical reaction scheme for intraprotein FAD^•–^–WH^•+^ radical pairs. The tertiary structure of the protein with the FAD cofactor and Trp tetrad shown for a European robin cryptochrome 4a (*Er*Cry4a) homology model.^71^ (B) The influence of dipolar coupling on the singlet yield anisotropies of the FAD^•–^–Z^•^ and FAD^•–^–WH^•+^ radical pairs. Anisotropy values are found in Table 1. Parameters are found in Table S3. An example script is found in the Supporting Information and both are available at examples/anisotropy_3d_polar_paper.py and examples/anisotropy_3d_polar_paper_2.py.

Kinetics are described with the exponential model and all parameters are provided in Table S3. The two radical pair systems show the importance of including more than one hyperfine coupling constant when modeling biochemical reactions at geomagnetic field strengths (50 μT) (Figure 6B), as it is well-known in the spin chemistry community that a one-proton radical pair overexaggerates the magnitude of the low field effect (LFE).^54^ This mechanism is believed to grant the magnetosensing ability of migratory animals and is different from the mechanism involved in higher magnetic fields.^72^ The plot.anisotropy_surface function was used to generate the plots.

The effect can be seen in both Figure 6B and Table 1, which produces values ΔΦ_*S*_ and  (using the utils.yield_anisotropy function) considerably larger than the more realistic model with a hyperfine coupling constant on each radical based on the crystal structure of the Cry homology model. Another important aspect is the addition of dipolar coupling, since now spherical averaging does not take place because of the restricted movement of the radical pair within the protein. As previously reported, in both cases the anisotropy is severely diminished.^73^

**Table 1:** Anisotropies for FAD^•–^–Z^•^ and FAD^•–^–WH^•+^ Radical Pair

Radical Pair	Dipolar	ΔΦ_*S*_	
FAD^•–^–Z^•^	no	0.249	0.468
	yes	0.015	0.025
FAD^•–^–WH^•+^	no	0.060	0.191
	yes	0.032	0.086

It has been proposed that a Cry binding partner (homo- or hetero-oligomerization) could amplify the compass sensing ability of migratory birds.^71^ Furthermore, other models involving radical scavengers have been shown to enhance this sensing ability in Cry simulations, however, experimental evidence is still required.^73^

### 3.5 Triplet Exciton Pairs in Crystals

In this example, we move away from radical pairs and into the realm of singlet fission (SF). The first direct experimental evidence for SF, in which one singlet exciton is split into two triplet excitons, was demonstrated with magnetic field effects on crystalline tetracene in the late 1960s.^74,75^ In recent times, SF has gained a lot of attention as a means of increasing the efficiency of solar cells.^76^ Moreover, the spin chemistry and spintronics communities have used magnetic field effects to better understand singlet fission in a range of materials and photovoltaic devices.^77,78^

**Figure 7:**
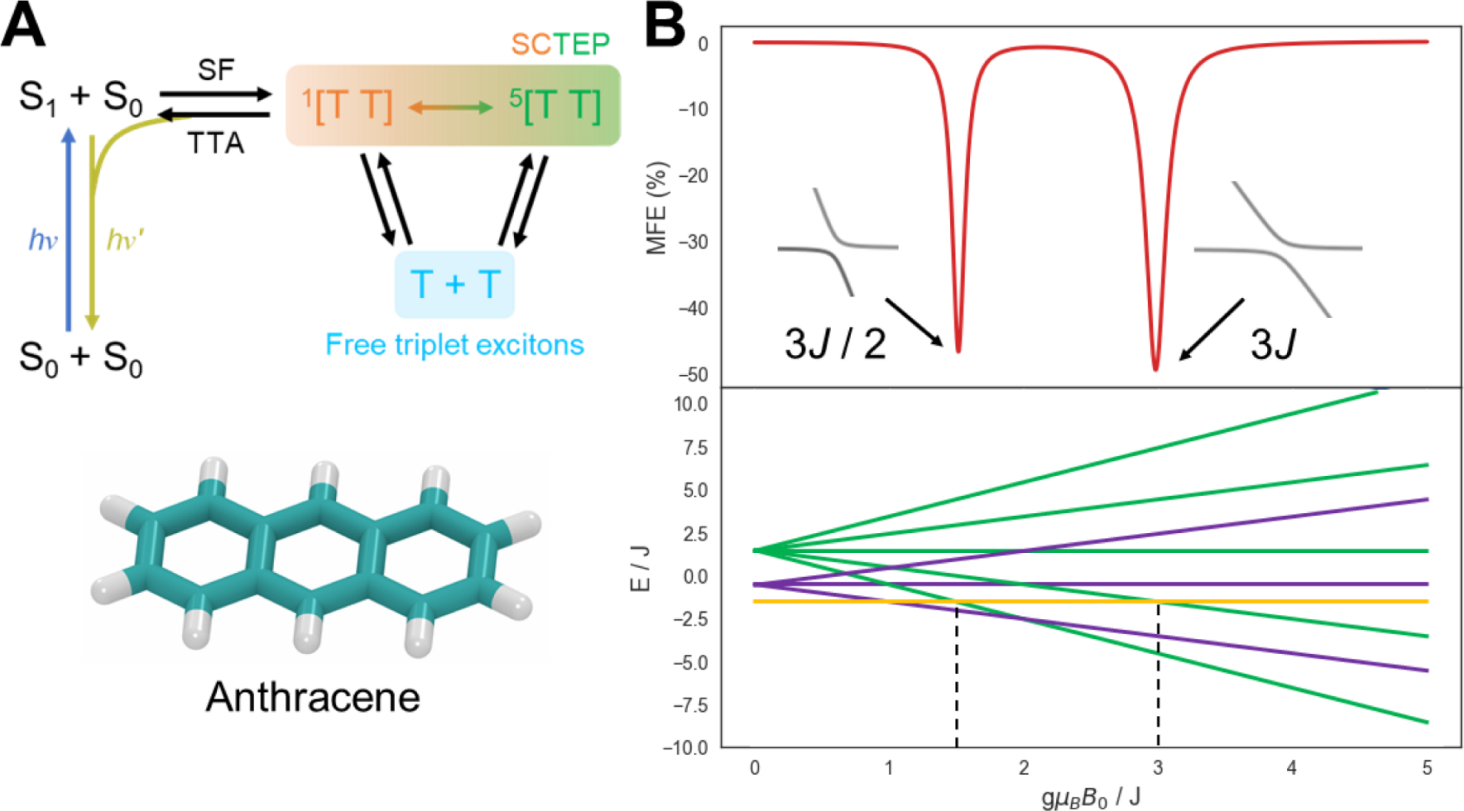
(A) Reaction scheme for the effect of a magnetic field on the delayed fluorescence of singlet fission in anthracene (shown below). The ZFS parameters are found in ref. (79). (B) Magnetic field effect on strongly coupled triplet pairs formed by singlet fission. The energy level diagram shows the singlet (*S* = 0, orange), triplet (*S* = 1, purple), and quintet (*S* = 2, green) manifolds, which are separated by the exchange interaction. A magnetic field effect is only observed at singlet-quintet level anticrossings. Parameters are found in Table S5. The script is found in the Supporting Information and is available at examples/sctep.py.

The example presented here considers SF in an anthracene crystal. Magnetic field effects are monitored by fluorescence (photoluminescence) under steady-state conditions (continuous excitation), as depicted in Figure 7. The triplet excitons are typically formed via photoinduced SF from the singlet excited state of anthracence, which can be shared with a neighboring ground-state anthracene. This can happen when the energy of the excitation source is approximately equal to or greater than twice the energy of a triplet state. Subsequently, a spin-correlated triplet exciton pair (SCTEP) is formed, by which zero-field splitting (ZFS) of the two triplets induces coherent interconversion between the singlet and quintet states (Figure 7). As with radical pairs, this singlet-quintet interconversion is affected by the magnitude and orientation of applied magnetic fields. Only exciton pairs in the overall singlet state may recombine to the singlet precursors via triplet–triplet annihilation (TTA), in which the singlet excited state may undergo radiative decay, emitting fluorescence.

For demonstration purposes, we assume that the SCTEP is in the strongly coupled regime where intertriplet exchange coupling dominates the intratriplet dipolar interaction (ZFS) (Figure 7). The spin Hamiltonian for the above steady-state experiment is given by , where  is given in eq S7, however, the nuclear spin term is neglected in this case. Hamiltonians  and  are described in eq S16. The SCTEP is constructed in the Zeeman basis by the following,

m = Triplet()
sim = LiouvilleSimulation(molecules=[m, m], basis=Basis.ZEEMAN)

The steady-state MARY simulation is acquired with experiments.steady_state_mary as shown in the following code snippet,

rhos, Phi_s = steady_state_mary(
  sim,
  obs=State.TP_SINGLET,
  Bs=Bs,
  D=D,
  E=E,
  J=J,
  theta=np.pi / 4,
  phi=0,
  kinetics=[Haberkorn(ktta, State.TP_SINGLET), HaberkornFree(kdiss)],
)

All parameters are given in Table S5.

The eigenvalues (or energies) for the triplet pair are plotted in the energy level diagram shown in Figure 7B. The equation of motion (eq S23) and the equation to obtain the fluorescence intensity under continuous photoexcitation (eq S24) are described in the Supporting Information. Figure 7B displays a magnetic field effect on an anthracene crystal under a strongly exchange-coupled limit and only exhibits a magnetic field effect at specific fields, which correlates to two level anticrossings between the singlet and quintet manifolds.

We have presented numerous examples involving spin-correlated radical and triplet pairs in various media, displaying the versatility of *RadicalPy*, and now we will move on to the conclusion section of the paper.

## 4 Conclusion

*RadicalPy* offers a comprehensive portfolio and a valuable addition to the spin chemistry, spintronics, and quantum biology communities, adding the missing pieces, the classical and semiclassical simulation methods, to complete the jigsaw puzzle. We have shown that *RadicalPy* can be used to simulate a selection of experiments with relatively little input from the user. The backend of the software deals with tedious computational aspects of simulations, thus alleviating the user of this burden.

The introduction of a new methodology for simulating experimental data, which includes aspects of classical, semiclassical, and quantum techniques, outperforms classical and quantum methods in both accuracy and performance (see Figures 1, 3, and 4). We foresee this method as a reliable and efficient way for experimentalists to reproduce their observations (if present) on radical/triplet pair phenomena, as well as other quantum systems, in soft and hard locales.

The software possesses not only a multitude of simulation techniques, but also numerous estimations (estimations.py) and utilities (utils.py) to aid the user. Estimation functions include *B*_1/2_,^60^*T*_1_/*T*_2_ relaxation rates,^80^ viscosity of aqueous/glycerol solutions,^81^ diffusion coefficients,^82^ dipolar and exchange interactions (for both molecules in solution^83^ and proteins),^84^ g-tensor relaxation rates,^85^ and rotational correlation times for molecules and proteins.^86^ Kinetic rate estimations are also supported, including spin dephasing,^32,33^ singlet–triplet mixing,^6^ electron transfer in proteins,^87^ singlet recombination,^88^ re-encounter rates,^89^ and triplet excited state relaxation rates.^90^ Utility functions include Lorentzian and *B*_1/2_ fitting for MARY spectra, magnetic flux density and spatial coordinate unit conversions, autocorrelation curves, and determination of spectral density frequencies.

The software also includes a novel rate equation solver and a Monte Carlo simulator for radical pairs in solution and micelles/vesicles. Combining Monte Carlo and autocorrelation methodologies offers an improved approach to estimate spin dephasing rates in micelles (see the Supporting Information). Further experimental simulations that are already implemented in the software include the nuclear spin polarization technique, chemically induced dynamic nuclear polarization (CIDNP)^3^ and modulated-MARY,^91^ which are found in the module experiments.py.

At present, the current version of the software only supports static magnetic field-based simulations; however, future versions of the software will support oscillating field-based simulations. Oscillating fields have been extensively used by the spin chemistry community to further provide evidence of quantum coherent phenomena in chemical and biological reactions.^6,92^ Resonance field experiments, reaction yield detected magnetic resonance (RYDMR)^93^ and oscillating magnetic field effects (OMFEs),^72^ are currently under development, which can be extended to the equivalent techniques electrically detected magnetic resonance (EDMR) and optically detected magnetic resonance (ODMR).^94,95^ Further theoretical resonant field-based approaches, such as γ-COMPUTE and local optimization theory, will be supported.^96,97^

The electron spin polarization method, chemically induced dynamic electron polarization (CIDEP)^1,2^ will appear in a future version of the software. We also aim to include photoselection and the switched external magnetic field (SEMF)^98^ technique to provide the user with a full suite of experimental methods and simulation techniques.

Both the functionality and the performance of the software are important for a smooth user experience. Documentation includes detailed descriptions of the software and references to the related paper.^99^ CI/CD (continuous integration and continuous deployment) is used during the software development process to automate tests that ensure that *RadicalPy* has no known bugs and that the documentation is always up-to-date. Spin chemistry tutorials using the *RadicalPy* software are also being prepared and will be distributed online as a learning and teaching tool. Furthermore, we foresee a graphical user interface (GUI) implemented in the software, alleviating all coding aspects and allowing those with a fear of coding to simulate their experimental results.

## Supporting Information Available

Descriptions of the quantum methods used in the present investigations; simple kinetic rate equation and Monte Carlo random walk examples; spin Hamiltonian memory allocation calculation; parameters and code listings for all figures presented in this investigation (PDF)
