# Supplementary material for: RadicalPy: A Tool for Spin Dynamics Simulations
Source: J Chem Theory Comput. 2024 Oct 29;20(21):9488–99. doi: 10.1021/acs.jctc.4c00887 (PMC11563354; doi:10.1021/acs.jctc.4c00887)
Supplement: Supplementary file 1 — ct4c00887_si_001.pdf [file ct4c00887_si_001.pdf]

RadicalPy: a tool for spin dynamics simulations

Lewis M. Antill*,†,¶ and Emil Vatai*,‡,¶

†*Department of Chemistry, University of Oxford, Physical and Theoretical Chemistry Laboratory, South Parks Road, Oxford OX1 3QZ, UK*

‡*High Performance Artificial Intelligence Systems Research Team, RIKEN Center for Computational Science, 7 Chome-1-26 Minatojima Minamimachi, Kobe, Hyogo 650-0047, Japan*

¶*These authors contributed equally*

E-mail: lewis.antill@chem.ox.ac.uk; emil.vatai@riken.jp

Supporting Information

S1 Classical simulations

S1.0.1 Chemical rate equations

For magnetic field effects on radical pairs to develop, the rates of reaction of the distinct spin states must be different. Radical pairs can undergo recombination to the ground state via back electron transfer or by a (non-)radiative transition back to the ground state (geminate recombination) with a rate constant, k_R. Only the singlet state can return to the ground state, and the quantum yields of this reaction are directly influenced by the effect of magnetic fields on the singlet character of the RP. Both spin states (singlet and triplet) of RPs can undergo spin decoherence at a rate, k_{Rlx}, or produce escape products at a rate, k_E, in solution-based systems, which typically give rise to long-lived species that can lead to large MFEs. These long-lived products also have an appreciable steady-state concentration as the photocycle accumulates over the course of multiple turnovers.

In 1984, Hayashi and Nagakura introduced the relaxation mechanism to describe the spin dynamics of radical pairs in the presence and absence of an external magnetic field with kinetic rate equations.[1] Figure S1 shows a simple schematic of the $B_0 = 0$ and $B_0 \gg$ HFC regimes. In the absence of an external magnetic field, the four spin states of the radical pair are degenerate and can undergo coherent singlet-triplet mixing (ST-mixing or spin-state mixing) at a rate, k_{ST}. In contrast, when the strength of an external magnetic field is much larger than the magnitudes of the hyperfine interactions of the radical species, the $T_{+/-}$ states become energetically separated from the S/T_0 states via the Zeeman interaction. Consequently, ST-mixing ceases between the S/T_0 and $T_{+/-}$ states, and spin relaxation occurs. These differences between the two cases lead to differences in the rates and yields of the chemical reaction.

The rate equations for a radical pair in zero field are given by,

$$
\begin{aligned}
\frac{d[S]}{dt} &= -(3k_{ST} + k_R + k_E)[S] + k_{ST}[T_+] + k_{ST}[T_0] + k_{ST}[T_-] \\
\frac{d[T_+]}{dt} &= -(2k_{ST} + k_E)[T_+] + k_{ST}[S] + k_{ST}[T_0] \\
\frac{d[T_0]}{dt} &= -(3k_{ST} + k_E)[T_0] + k_{ST}[S] + k_{ST}[T_+] + k_{ST}[T_-] \\
\frac{d[T_-]}{dt} &= -(2k_{ST} + k_E)[T_-] + k_{ST}[S] + k_{ST}[T_0].
\end{aligned}
\tag{S1}
$$

The code is written as,

```
off[S] = {S: -(3 * kst + kr + ke), Tp: kst, T0: kst, Tm: kst}
off[Tp] = {Tp: -(2 * kst + ke), S: kst, T0: kst}
off[T0] = {T0: -(3 * kst + ke), S: kst, Tp: kst, Tm: kst}
off[Tm] = {Tm: -(2 * kst + ke), S: kst, T0: kst}
```

And similarly when the external magnetic field strength is far greater than the hyperfine interactions in the radical pair,

$$
\begin{aligned}
\frac{d[S]}{dt} &= -(k_{ST} + 2k_{Rlx} + k_R + k_E)[S] + k_{Rlx}[T_+] + k_{ST}[T_0] + k_{Rlx}[T_-] \\
\frac{d[T_+]}{dt} &= -(2k_{Rlx} + k_E)[T_+] + k_{Rlx}[S] + k_{Rlx}[T_0] \\
\frac{d[T_0]}{dt} &= -(k_{ST} + 2k_{Rlx} + k_E)[T_0] + k_{ST}[S] + k_{Rlx}[T_+] + k_{Rlx}[T_-] \\
\frac{d[T_-]}{dt} &= -(2k_{Rlx} + k_E)[T_-] + k_{Rlx}[S] + k_{Rlx}[T_0].
\end{aligned}
\tag{S2}
$$

The corresponding code,

```
on[S] = {S: -(kst + 2 * krlx + kr + ke), Tp: krlx, T0: kst, Tm: krlx}
on[Tp] = {Tp: -(2 * krlx + ke), S: krlx, T0: krlx}
on[T0] = {T0: -(kst + 2 * krlx + ke), S: kst, Tp: krlx, Tm: krlx}
on[Tm] = {Tm: -(2 * krlx + ke), S: krlx, T0: krlx}
```

One can select the initial states (and their initial populations) for a triplet born radical pair and simulation time with the following:

```
initial_states = {Tp: 1 / 3, T0: 1 / 3, Tm: 1 / 3}
```

The class `classical.RateEquations` transforms the differential rate equations into a propagator matrix, which can then be used to calculate the time evolution of the initial states with the `time_evolution` function,

```
time = np.linspace(0, 1e-6, 10000)


roff = RateEquations(off)
ron = RateEquations(on)
result_off = RateEquations.time_evolution(roff, time, initial_states)
result_on = RateEquations.time_evolution(ron, time, initial_states)
```

Finally, we select the four states of the radical pair (`keys`) to acquire ΔA in the presence and absence of an external magnetic field and calculate the magnetic field effect by taking the difference of the two ($\Delta\Delta A$), as shown in Figure S1.

```
keys = [S, Tp, T0, Tm]
rp_field_off = result_off[keys]
rp_field_on = result_on[keys]
rp_delta_delta_A = rp_field_on - rp_field_off
```

The rate constants are input using the `classical.Rate` function, which also allows one to input the LaTeX syntax,

```
ke = Rate(1e6, "k_{E}")
kst = Rate(8e7, "k_{ST}")
krlx = Rate(2e6, "k_{Rlx}")
kr = Rate(1e8, "k_{R}")
```

The code for `off` and `on` are converted to LaTeX with the following,

```
print(latex_eqlist_to_align(latexify(off)))
print(latex_eqlist_to_align(latexify(on)))
```

Which produce rate equations ((S1)-(S2)).

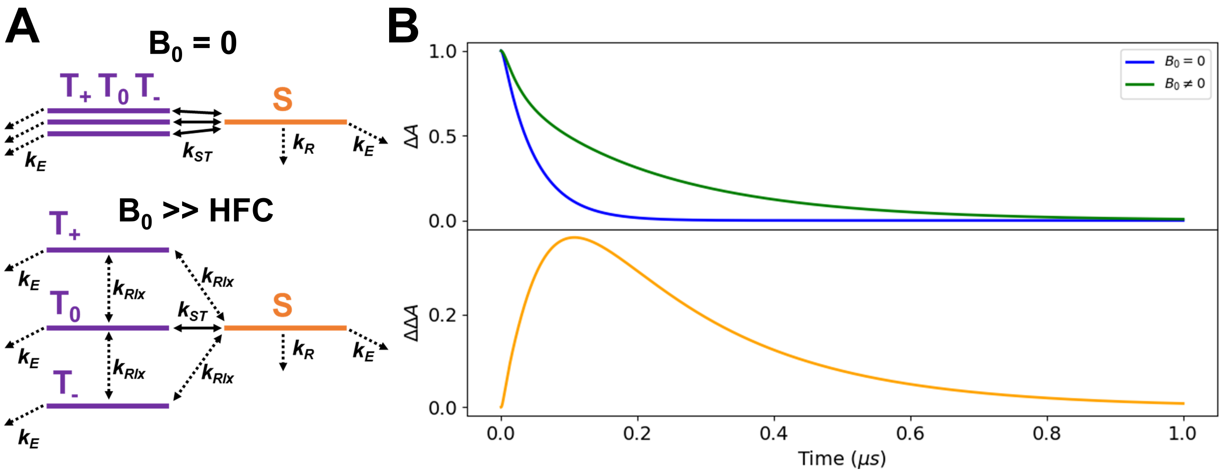

Figure S1: **A** (top): Reaction scheme for a radical pair in the absence of an external magnetic field. S denotes the singlet state, T_+, T_0, T_- are the triplet states, k_E, k_R, k_{ST}, represent the rates for escape products, singlet recombination, and singlet-triplet mixing, respectively. **A** (bottom): Reaction scheme for a radical pair in the presence of an external magnetic field with magnitude far greater than that of the hyperfine interactions of the radical pair. k_{Rlx} is the spin relaxation rate. **B** (top): ΔA absorption kinetics in the presence (green) and absence (blue) of an external magnetic field. **B** (bottom): $\Delta\Delta A$ (MFE) time profile for a triplet born radical pair. Kinetic rate constants are $k_E = 1 \times 10^6$ s^{-1}, $k_R = 1 \times 10^8$ s^{-1}, $k_{Rlx} = 2 \times 10^6$ s^{-1}, and $k_{ST} = 8 \times 10^7$ s^{-1}.

S1.0.2 Monte Carlo random walk

In solution, a typical lifetime of radical pairs of ≈ 10 ns usually competes with diffusive dissociation (free radical formation) of RPs.[2] However, if the RPs are confined in some way, long-lived radical pairs of $\gg 10$ ns are observed and consequently produce a larger MFE. Typical reaction environments, where the RPs are encapsulated or confined in chemical cages, include micelles,[3,4] vesicles,[5] ionic liquids,[6] and proteins.[7]

One method for simulating molecular diffusion and different reaction environments is the Monte Carlo random walk. The Monte Carlo method uses random sampling in the direction in which the radical moves in the three-dimensional space.[8,9] The Wiener process is a stochastic process that provides a behaviour similar to Brownian motion and can be used to describe molecular diffusion.

The diffusional motion of the radical pair will be considered as random jumps between

two points separated by a distance Δr, given by $\Delta r = \sqrt{6 D_{AB} \Delta t}$, where Δt is the time interval and D_{AB} is the mutual diffusion coefficient of the radical pair. This equation for calculating the mean distance corresponds to the function `classical.get_delta_r`. The Monte Carlo random walk is achieved with the function `classical.randomwalk_3d`, which provides radical pair diffusion simulations in both solution and encapsulated environments.

The following example describes a radical pair encapsulated in a reverse micelle, e.g., water and sodium bis(2-ethylhexyl) sulfosuccinate (AOT), where the size of the micelle is related to the volume of water. The water-to-AOT ratio, w_0, is given by $w_0 = \frac{[H_2O]}{[AOT]}$. Where the higher the value of w_0 the larger the size of the reverse micelle. Figure S2 shows a Monte Carlo random walk simulation for a radical pair encapsulated in an AOT reverse micelle with a radius of 10 Å (`r_max`, $w_0 = 3$) and a distance from the closest approach of 5 Å (`r_min`). The code for 600×40 ns trajectories with $D_{AB} = 1 \times 10^{-6}$ cm s^{-1} and $\Delta t = 40$ ps are given by,

```
np.random.seed(42)
t = np.arange(0, 40e-9, 40e-12)
r_min = 5e-10 / 2
r_max = 10e-10
r = (r_min) + np.random.sample() * ((r_max) - (r_min))
x0, y0, z0 = r, 0, 0
mutual_diffusion = 1e-6 / 10000
N = 600
```

Δr and the Monte Carlo random walk are calculated with the following,

```
delta_r = rp.classical.get_delta_r(mutual_diffusion, t[1] - t[0])
dist, ang = np.zeros([N, len(t)]), np.zeros([N, len(t)])

for i in tqdm.tqdm(range(0, N)):
    pos, dist[i], ang[i] = rp.classical.randomwalk_3d(
        len(t), x0, y0, z0, delta_r, r_min, r_max
    )
```

The plot in Figure S2A is easily constructed with the following,

```
rp.plot.monte_carlo_caged(pos, r_max)
```

The fluctuation of spin-spin interactions induced by diffusional motion can be estimated for the above result as they are dependent on the separation, r, between the radical pair (Figure S2B). The distance dependence of the exchange coupling, J, is given by

$$J(r) = -J_0 e^{-\alpha(r(t) - r_{min})}, \tag{S3}$$

where $J_0 = -570$ mT and $\alpha = 2$ Å^{-1}, which are common values for neutral radical pairs.[9] The corresponding code,

```
J_all = rp.estimations.exchange_interaction_in_solution_MC(dist)
```

We include a method for estimating singlet-triplet dephasing, k_{STD}, relaxation rates from Monte Carlo random walk calculations. The spin dephasing rates are estimated with the `estimations.k_STD` function and are calculated by the following,

$$k_{\mathrm{STD}} = 4\tau_c \, \mathrm{Var}[J]. \tag{S4}$$

Where τ_c is the correlation time which characterises the fluctuations in the radical pair separation, r, and Var is the variance.[10] This methodology agrees particularly well with the analytical formula developed by Shushin[11] (Fig. S2C). However, spin dephasing rates for reverse micelles with $\omega_0 > 25$ estimated with the Monte Carlo method produce values an order of magnitude smaller than the Shushin approach, which is given by,

$$k_{\mathrm{STD}} = \frac{4\pi D l}{V}. \tag{S5}$$

Where D is the mutual diffusion coefficient, V is the volume of the microreactor, and l is the general analytical expression of the complex spin-exchange relaxation radius.[11] The

above equation (S5) is supported by *RadicalPy* with the `estimations.k_STD_microreactor` function. The values obtained above $\omega_0 > 25$ are overestimated by the Shushin method, since the radius of the reverse micelle is greater than 4 nm, where the exchange interaction is negligible. Our Monte Carlo method estimates the exchange interaction to be ≈ 20 µT and gives more reasonable values of k_{STD} on the $\times 10^4 - \times 10^5$ s^{-1} order compared to k_{STD} on the $\times 10^5 - \times 10^6$ s^{-1} order estimated by the Shushin approach (Fig. S2C). An advantage of equation (S4) is that it can be used to analyse fluctuations in radical pair separation in molecular dynamics (MD) simulations.[10] Figure 5 in the main text demonstrates this analysis on the molecular dynamics of a radical pair in an AOT reverse micelle.

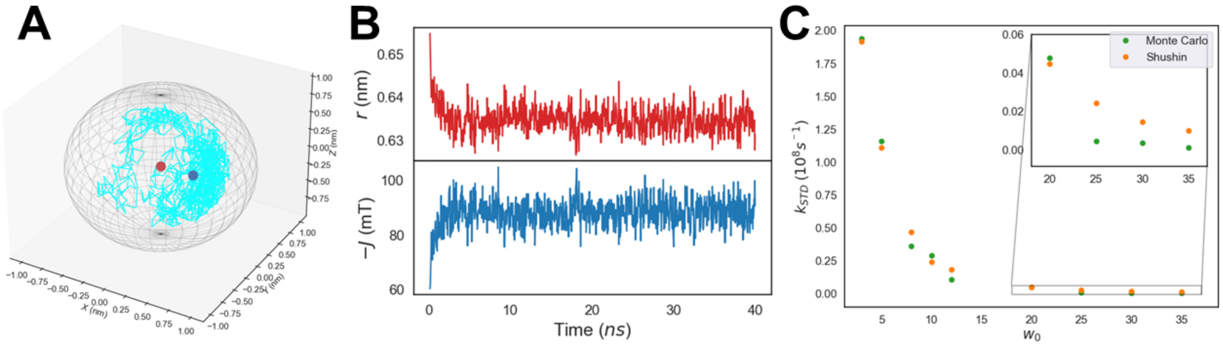

Figure S2: **A**: A single Monte Carlo random walk simulation for a radical pair encapsulated in a $w_0 = 3$ reverse micelle. $D_{AB} = 1 \times 10^{-6}$ cm s^{-1} and $\Delta t = 40$ ps. **B**: Time evolution of the mean inter-radical separation (r) and exchange interaction (J) for 600 trajectories. **C**: Comparison of Monte Carlo random walk and Shushin[11] methodologies for estimating J-modulation induced spin dephasing rates as a function of the reverse micelle size.

S1.1 Quantum simulations

A tedious and often error-prone process of creating spin chemistry simulations is in specifying the simulated molecules and their nuclei; therefore, *RadicalPy* includes a database to eliminate this burden. To reduce redundancy and maximise flexibility, the database is divided into two parts: the *isotope database* containing the spin multiplicity and the magnetogyric ratio for 293 isotopes,[12] and the *molecules database* compiled from various in-house DFT calculations.[13,14] The isotope data are stored in a single JSON file, whereas molecules are stored in separate files, listing information about the nuclei of each molecule, including the isotope (referencing the other database), the hyperfine coupling constant, and the nuclei identifier string in the molecule. Having a separate database for isotopes makes it possible to construct "custom" molecules, i.e., the user can create a molecule simply by listing the isotopes and the corresponding hyperfine coupling constants. Currently, *RadicalPy* houses a limited number of molecules that are easily expandable and growing (and we encourage community contributions).

In *RadicalPy*, a radical pair is represented as a pair of `Molecule` objects each specifying the multiplicities, magnetogyric ratios, and hyperfine coupling constants of the nuclei of the molecule for the simulation of choice. The construction of such molecules can be easily accomplished using the `fromdb` class method, which is the interface to the molecule database. These `Molecule` objects are then used to create a simulation (`sim`) object.

The class name `HilbertSimulation` refers to the use of Hilbert space for simulations. For simulations in Liouville space, the class `LiouvilleSimulation` can be used. Using JSON files is a convenient way to store information in both human- and machine-readable formats, and the clean separation of the database interface provides the possibility of swapping out the JSON files for a more sophisticated backend, such as an online portal that would allow users to upload custom molecules.

S1.1.1 Total spin Hamiltonian

The numerous interaction terms in the radical pair are described by the total spin Hamiltonian, which govern the time dependence of the spin dynamics of the radical pair,

$$\hat{H}_{\text{total}} = \hat{H}_Z + \hat{H}_H + \hat{H}_J + \hat{H}_D. \tag{S6}$$

Internal interactions in the radical are described by the Zeeman ($\hat{H}_Z$) and hyperfine ($\hat{H}_H$) terms. Here, the exchange ($\hat{H}_J$) and dipolar ($\hat{H}_D$) terms represent the electron-electron coupling terms.

The Zeeman interaction describes the interaction between the magnetic dipole moment associated with electron, i, and nucleus, j, spins and an external magnetic field,

$$\begin{aligned} \hat{H}_Z &= \omega_0^{(i)} \cdot \hat{\mathbf{S}}_i + \omega_0^{(j)} \cdot \hat{\mathbf{I}}_j \\ &= -\gamma_e^{(i)} B_0 \cdot \hat{\mathbf{S}}_i + \pm\gamma_n^{(j)} B_0 \cdot \hat{\mathbf{I}}_j \\ &= \frac{-g_e^{(i)} \mu_B B_0}{\hbar} \cdot \hat{\mathbf{S}}_i + \frac{\pm g_n^{(j)} \mu_n B_0}{\hbar} \cdot \hat{\mathbf{I}}_j. \end{aligned} \tag{S7}$$

Where ω_0 is the Larmor frequency; g_e and g_n represent Landé g-factors; μ_B and μ_n define the Bohr magneton and nuclear magneton, respectively; γ_e and γ_n denote magnetogyric ratios; B_0 is the corresponding external magnetic field strength; $\hat{\mathbf{S}}$ and $\hat{\mathbf{I}}$ are the vectors of Cartesian electron and nuclear spin operators, $\{\hat{S}_x, \hat{S}_y, \hat{S}_z\}$ and $\{\hat{I}_x, \hat{I}_y, \hat{I}_z\}$, respectively.

The second term describes the hyperfine interaction between unpaired electron spins and surrounding nuclear spins. The isotropic (Fermi contact) and anisotropic (dipolar) interactions are combined to give the hyperfine interaction between the unpaired electron in radical, i, and nucleus, j, in the same molecule, where the total hyperfine interaction is the sum of the individual hyperfine terms (where i iterates over each radical, and j sums over

the number of nuclei associated with radical i),

$$\hat{H}_H = \sum_i \sum_j \hat{\mathbf{S}}_i \cdot A_{ij} \cdot \hat{\mathbf{I}}_{ij}. \tag{S8}$$

The direction dependence and strength of the hyperfine interaction are contained in the hyperfine tensor A_{ij}; $\hat{\mathbf{I}}_{ij}$ is the spin operator for the nucleus, j, in radical, i. For the isotropic hyperfine interaction, a, the following Hamiltonian is used,

$$\hat{H}_H = a\hat{\mathbf{S}} \cdot \hat{\mathbf{I}}. \tag{S9}$$

The exchange interaction details the overlap of the spatial wavefunctions of the two electrons, which is effective at short radical-radical distances. The Hamiltonian is given by,

$$\hat{H}_J = -J\Big(2\,\hat{\mathbf{S}}_1 \cdot \hat{\mathbf{S}}_2 + \frac{1}{2}\hat{E}\Big). \tag{S10}$$

Here, $\hat{E}$ is the identity matrix. One can assume that the magnitude of the exchange coupling constant, J, decreases exponentially with increasing radical pair separation, r, and is independent of the relative radical orientations,

$$J(r) = J_0 e^{-r/r_J}, \tag{S11}$$

where J_0 is the strength of the interaction and r_J is the range parameter.

The anisotropic dipolar coupling term captures the interaction of each electron with the magnetic field emanating from the other unpaired electron,

$$\hat{H}_D = \hat{\mathbf{S}}_1 \cdot D \cdot \hat{\mathbf{S}}_2. \tag{S12}$$

The dipolar interaction tensor, D, is given by,[15]

$$D = \frac{2}{3}\begin{pmatrix} -1 & 0 & 0 \\ 0 & -1 & 0 \\ 0 & 0 & 2 \end{pmatrix} \gamma_e \frac{3 g_e \mu_B \mu_0}{8\pi r^3}. \tag{S13}$$

The isotropic dipolar Hamiltonian is given by,

$$\hat{H}_D = \frac{2}{3} D\Big(3\hat{\mathbf{S}}_{1z} \cdot \hat{\mathbf{S}}_{2z} - \hat{\mathbf{S}}_1 \hat{\mathbf{S}}_2\Big) \tag{S14}$$

$$D = \gamma_e \frac{3 g_e \mu_B \mu_0}{8\pi r^3}. \tag{S15}$$

where μ_0 is the vacuum magnetic permeability. D scales with a r^{-3} dependence as opposed to J, which decays exponentially and, as a result, dipolar coupling is dominant at larger radical pair separations.

The zero-field splitting and exchange interaction Hamiltonians for the triplet exciton pair, are given by,

$$\begin{aligned} \hat{H}_{\text{ZFS}} &= \sum_{i=1}^{2} D\Big(\hat{\mathbf{S}}_{i,z}^2 - \frac{1}{3}(\hat{\mathbf{S}}_{i,x}^2 + \hat{\mathbf{S}}_{i,y}^2 + \hat{\mathbf{S}}_{i,z}^2)\Big) + E\Big(\hat{\mathbf{S}}_{i,x}^2 - \hat{\mathbf{S}}_{i,y}^2\Big) \\ \hat{H}_J &= -J\Big(\hat{\mathbf{S}}_1 \cdot \hat{\mathbf{S}}_2 + \frac{1}{2}\hat{E}\Big). \end{aligned} \tag{S16}$$

Total spin Hamiltonian in *RadicalPy* The total spin Hamiltonian (S6) and its components are implemented as methods of the `sim` object and can be invoked as follows:

```
H = sim.total_hamiltonian(B0=0, D=0, J=0)
```

Since the total spin Hamiltonian is a sum of the Zeeman, hyperfine, exchange, and dipolar Hamiltonians, the parameters of the `total_hamiltonian` are the union of the parameters of the component methods.

The parameter `B0` is the external magnetic field strength, B_0, of the Zeeman Hamiltonian of (S7).

The dipolar Hamiltonian, `D`, can be the dipolar interaction tensor (S13) or constant (S15), in which case the anisotropic equation (S12) or the isotropic equation (S14) is used to calculate H_D, respectively. Finally, `J` is the exchange coupling constant (S11) of the exchange Hamiltonian (S10). The module `estimations` of *RadicalPy* contains various functions to calculate D and J.

In addition to the parameters shown in the code snippet above, the `total_hamiltonain` method also has optional parameters. The parameters `theta` and `phi` are the rotation angles between the external magnetic field and the fixed molecule used for the anisotropy experiments. The optional parameter `hfc_anisotropy` is set by default to *false*, in which case the isotropic equation (S9) is used, and when set to *true*, the anisotropic equation (S8) is used (assuming that hyperfine tensor, A_{ij}, is available for all nuclei).

S1.1.2 The equation of motion

The density matrix formalism is a common tool in spin quantum mechanics, as it allows one to describe and generalise large spin systems and their ensembles. Density matrices embody the idea of mixed states and allow us to describe the spin states and dynamics of radical pair reactions.

For a system with n, m possible states, the density operator, $\hat{\rho}$, is defined as $\hat{\rho} = \sum_{n,m} c_n c_m^* \left|\psi_n\right\rangle \left\langle\psi_m\right|$. The density matrix, ρ, evolves over time according to the Liouville-von Neumann equation (in Hilbert space) where $\frac{\partial \hat{\rho}}{\partial t} = -i[\hat{H}, \rho]$, with $\hat{H}$ being the time-dependent spin Hamiltonian. Integration of the above equation produces,

$$\rho(t) = \mathbf{U}\rho_0\mathbf{U}^*, \tag{S17}$$

the unitary operators are,

$$\mathbf{U} = \exp(-i\hat{H}t) \tag{S18}$$

$$\mathbf{U}^* = \exp(+i\hat{H}t). \tag{S19}$$

In Liouville space the Liouville-von Neumann equation takes the form,

$$\frac{\partial \hat{\rho}}{\partial t} = -i\hat{\hat{L}}(t)\rho(t), \tag{S20}$$

where the Liouvillian, $\hat{\hat{L}}$, is obtained by the following commutator relationship,

$$\hat{\hat{L}} = \hat{H} \otimes \hat{E} - \hat{E} \otimes \hat{H}^T, \tag{S21}$$

and integration of the above equation yields,

$$\rho(t) = \exp(\hat{\hat{L}}t)\rho_0 = \mathbf{U}\rho_0. \tag{S22}$$

In radical pair spin dynamics, we are generally interested in a particular state (observable) of the system, *e.g.,* the singlet state. Therefore, we need to project out the desired component of the density matrix. The projection of $\rho(t)$ onto a state n is given by the trace of the product of the projection operator, $\hat{Q}_n$, with the density matrix giving the expectation value, $\langle \hat{Q}_n \rangle$, explicitly $\langle \hat{Q}_n \rangle = \mathrm{Tr}\big[\hat{\rho}\,\hat{Q}_n\big]$. Here Tr is the trace. The projection operators, $\hat{Q}_n$, for radical pairs are described below.

S1.1.3 Equation of motion for continuous excitation

The equation of motion for continuous photoexcitation, where $\frac{d\rho}{dt} = 0$, is given by,[16]

$$\begin{aligned} -i\hat{\hat{\mathbf{H}}}\rho - \hat{\hat{\mathbf{K}}}_S - \hat{\hat{\mathbf{K}}}_F &= k_{\mathrm{SF}}\hat{\mathbf{Q}}_{\mathrm{TP}_S} \\ \hat{\hat{\mathbf{L}}}\rho &= k_{\mathrm{SF}}\hat{\mathbf{Q}}_{\mathrm{TP}_S}. \end{aligned} \tag{S23}$$

Where $\hat{\hat{\mathbf{K}}}_S$ is the Haberkorn superoperator for the SCTEP returning to S_0 and S_1 via triplet-triplet annihilation, at a rate k_{TTA}. $\hat{\hat{\mathbf{K}}}_F$ describes the dissociation of the SCTEP from free triplet excitons. k_{SF} is the zeroth order rate constant (due to continuous excitation) for the formation of the SCTEP and $\hat{\mathbf{Q}}_{\mathrm{TP}_S}$ is the singlet projection operator for the triplet pair.

The fluorescence intensity, I_F, is obtained by,[16]

$$I_F = k_{SF}k_{TTA}\hat{\mathbf{Q}}^{\dagger}_{\mathrm{TP}_S}\hat{\hat{\mathbf{L}}}^{-1}\hat{\mathbf{Q}}_{\mathrm{TP}_S}. \tag{S24}$$

The projection operator $\hat{\mathbf{Q}}_{\mathrm{TP}_S}$ is described in equation (S26).

S1.1.4 Projection operators

The projection operators, $\hat{Q}_n$, for radical pairs are described as follows,

$$\begin{aligned} \hat{Q}_S =& \frac{1}{4}\hat{E} - \hat{\mathbf{S}}_1 \cdot \hat{\mathbf{S}}_2 \\ \hat{Q}_T =& \frac{3}{4}\hat{E} + \hat{\mathbf{S}}_1 \cdot \hat{\mathbf{S}}_2 \\ \hat{Q}_{T_+} =& \big(2\hat{\mathbf{S}}^2_{1z} + \hat{\mathbf{S}}_{1z}\big) \odot \big(2\hat{\mathbf{S}}^2_{2z} + \hat{\mathbf{S}}_{2z}\big) \\ \hat{Q}_{T_0} =& \frac{1}{4}\hat{E} + \hat{\mathbf{S}}_{1x} \cdot \hat{\mathbf{S}}_{2x} + \hat{\mathbf{S}}_{1y} \cdot \hat{\mathbf{S}}_{2y} - \hat{\mathbf{S}}_{1z} \cdot \hat{\mathbf{S}}_{2z} \\ \hat{Q}_{T_-} =& \big(2\hat{\mathbf{S}}^2_{1z} - \hat{\mathbf{S}}_{1z}\big) \odot \big(2\hat{\mathbf{S}}^2_{2z} - \hat{\mathbf{S}}_{2z}\big). \end{aligned} \tag{S25}$$

where $\odot$ denotes element-wise multiplication. The projection operator for the singlet state of a triplet exciton pair (as used in Equation (S24)), is given by,

$$\begin{aligned} \hat{\mathbf{S}}^2 &= \hat{\mathbf{S}}_{1x}^2 + \hat{\mathbf{S}}_{1y}^2 + \hat{\mathbf{S}}_{1z}^2 + \hat{\mathbf{S}}_{2x}^2 + \hat{\mathbf{S}}_{2y}^2 + \hat{\mathbf{S}}_{2z}^2 + 2\Big(\hat{\mathbf{S}}_{1x} \cdot \hat{\mathbf{S}}_{2x} + \hat{\mathbf{S}}_{1y} \cdot \hat{\mathbf{S}}_{2y} + \hat{\mathbf{S}}_{1z} \cdot \hat{\mathbf{S}}_{2z}\Big) \\ \hat{\mathbf{Q}}_{\mathrm{TP}_S} &= \frac{1}{12}\Big(\hat{\mathbf{S}}^2 - 6\hat{\mathbf{E}}\Big) \cdot \Big(\hat{\mathbf{S}}^2 - 2\hat{\mathbf{E}}\Big) \end{aligned} \tag{S26}$$

The equation of motion in *RadicalPy* The following code snippet computes the time evolution (for 2 μs) of the density matrix, ρ,

```
time = np.arange(0, 2e-6, 5e-9)
rhos = sim.time_evolution(State.SINGLET, time, H)
```

The parameters are the initial state (`State.SINGLET`) of the density matrix, an array of (uniform) time steps (in this instance 5 ns) (usually from `np.arange` or `np.linspace`) and the Hamiltonian. Internally `time_evolution` calls a `unitary_propagator` method that, in Hilbert space, calculates $\mathbf{U}$ and $\mathbf{U}^*$ from (S18) and (S19), which is plugged into (S17) to generate the time evolution of ρ by iterating the `propagate` method on the density matrix, starting with the output of the `projection_operator` that constructs one of the operators from (S25).

When switching from Hilbert space to Liouville space, the user only needs to use the `LiouvilleSimulation` class instead of the `HilbertSimulation` class when instantiating the `sim` object (as described in Section S1.1). Since the latter class is derived from the former, the sim object inherits all the members and behaviour of the `HilbertSimulation` class. In this case, the `total_hamiltonian()` method invokes the `convert(H)` method, which converts the Hamiltonian to a Liouvillian according to (S21) (for the separate components of the total Hamiltonian, such as H_Z, H_H, H_J and H_D, the `convert(H)` method must be called manually). The relevant methods are overloaded and ensure correct calculations for Liouville space simulations, *e.g.*, `unitary_propagator` and `propagate` implement (S22) and (S20), resulting in `time_evolution` performing the correct calculations.

S1.1.5 Radical pair anisotropy

When an immobilised radical pair is considered, the anisotropic spin interactions are dependent on the relative orientations of the molecules. The spin Hamiltonian then becomes dependent on the angles, θ and ϕ, between the external magnetic field and the fixed molecule. The Zeeman Hamiltonian takes the form,

$$\begin{aligned}\hat{H}_Z = \omega_0^{(i)} \cdot \left[\hat{S}_x \sin\theta \cos\phi + \hat{S}_y \sin\theta \sin\phi + \hat{S}_z \cos\theta\right] \\ +\omega_0^{(j)} \cdot \left[\hat{I}_x \sin\theta \cos\phi + \hat{I}_y \sin\theta \sin\phi + \hat{I}_z \cos\theta\right].\end{aligned}$$

The product yield of the radical pair reaction is given by,

$$\Phi_n(\theta, \phi) = k \int_0^\infty \hat{Q}_n(t) dt. \tag{S27}$$

Here, k is the first-order rate constant, and $\hat{Q}_n$ denotes the projection operator. The spherically averaged product yield is calculated using a trapezoidal rule approximation of the integral,[17]

$$\langle \Phi_n \rangle = \frac{1}{4\pi} \int_0^{2\pi} \int_0^{\pi} \Phi_n(\theta, \phi) \sin\theta \, d\theta \, d\phi. \tag{S28}$$

The anisotropy of the product yield can be evaluated by the following,[18]

$$\Delta\Phi_n = \max[\Phi_n] - \min[\Phi_n] \tag{S29}$$

$$\Gamma_n = \frac{\Delta\Phi_n}{\langle \Phi_n \rangle}. \tag{S30}$$

$\Delta\Phi_n$ is a commonly used metric of anisotropy used in quantum biology. Γ_n is generally used to evaluate the magnetic compass, where the higher the value, the better the compass.[18]

Radical pair anisotropy in *RadicalPy* The anisotropic version of the Zeeman Hamiltonian (S27) is automatically used if the arguments `theta` and `phi` are specified when calling

the `total_hamiltonian` (see Section S1.1.1). The function `product_yield` calculates the product yield (S27) and the sum of the product yield. The values of $\Delta\Phi_n$ (S29) and Γ_n (S30) are calculated using `yield_anisotropy` and `spherical_average` to calculate the spherical average (S28) (both from the module `utils`).

S1.1.6 Incoherent processes

To paint a more complete picture of the real physical processes involved in radical pair chemical reactions one must include kinetics and relaxation. Collectively, they are known as incoherent processes. In essence, kinetics and relaxation drive chemical reactions back to thermal equilibrium. When considering biological reactions, where the environment is 37 °C and "squishy", including the relevant kinetics and relaxation descriptions is essential to understand the key mechanisms involved. *RadicalPy* supplies various kinetics and relaxation (super)operators, which are described in the following sections.

S1.1.7 Kinetics (super)operators

The exponential model assumes that after a first re-encounter, radicals either react or diffuse apart forever,

$$f(t) = ke^{-kt}, \tag{S31}$$

Where k is a phenomenological rate constant.

Chemical reactions in Liouville space can be modelled with Haberkorn superoperators $\hat{\hat{K}}$.[19] Spin-selective formation of either the singlet or triplet state products are given by,

$$\hat{\hat{K}}_S = -\frac{k_S}{2}\Big(\hat{Q}_S \otimes \hat{E} + \hat{E} \otimes \hat{Q}_S\Big) \tag{S32}$$

$$\hat{\hat{K}}_T = -\frac{k_T}{2}\Big(\hat{Q}_T \otimes \hat{E} + \hat{E} \otimes \hat{Q}_T\Big), \tag{S33}$$

where k_S and k_T are the first-order rate constants for singlet and triplet pathways, respec-

tively. Singlet and triplet states that diffuse apart, with the same first-order rate constant k_F, to form free radicals, are described by,

$$\hat{\hat{K}}_\mathrm{F} = k_\mathrm{F} \hat{E} \otimes \hat{E}. \tag{S34}$$

An alternative approach based on quantum measurement theory, known as the Jones-Hore master equation, is the same as the Haberkorn model but includes an additional singlet-triplet dephasing term,[20]

$$\hat{\hat{K}}_\mathrm{JH} = \hat{\hat{K}}_S + \hat{\hat{K}}_T + \frac{k_S + k_T}{2}\Big(\hat{Q}_S \otimes \hat{Q}_T + \hat{Q}_T \otimes \hat{Q}_S\Big). \tag{S35}$$

S1.1.8 Relaxation superoperators

Spin relaxation can have a profound effect on radical pair reactions. Numerous explicit models are reported in the literature, some of which are supported by *RadicalPy* and are described below. Singlet-triplet dephasing arises from modulation of the electron-exchange interaction.[11] The relaxation superoperator, with a dephasing rate, k_STD, is written as,

$$\hat{\hat{R}}_\mathrm{STD} = k_\mathrm{STD}\Big(\hat{Q}_S \otimes \hat{Q}_T + \hat{Q}_T \otimes \hat{Q}_S\Big). \tag{S36}$$

Random modulation of the anisotropic dipole-dipole spin interaction can be modelled by the triplet-triplet dephasing superoperator,[21]

$$\hat{\hat{R}}_\mathrm{TTD} = k_\mathrm{TTD}\Big(\hat{Q}_{T_+} \otimes \hat{Q}_{T_-} + \hat{Q}_{T_-} \otimes \hat{Q}_{T_+} + \hat{Q}_{T_0} \otimes \hat{Q}_{T_-} + \hat{Q}_{T_-} \otimes \hat{Q}_{T_0} + \hat{Q}_{T_0} \otimes \hat{Q}_{T_+} + \hat{Q}_{T_+} \otimes \hat{Q}_{T_0}\Big), \tag{S37}$$

where k_TTD is the dephasing rate. $\hat{\hat{R}}_\mathrm{STD}$ and $\hat{\hat{R}}_\mathrm{TTD}$ are generally implemented together when simulating dipolar coupling modulation. An alternative dipolar coupling modulation

relaxation superoperator was recently reported,[10] with relaxation rate, k_{D},

$$\hat{\hat{R}}_{\mathrm{D}} = k_{\mathrm{D}}\Big(\frac{1}{9}\hat{Q}_S \otimes \hat{Q}_{T_+} + \frac{1}{9}\hat{Q}_{T_+} \otimes \hat{Q}_S + \frac{1}{9}\hat{Q}_S \otimes \hat{Q}_{T_-} + \frac{1}{9}\hat{Q}_{T_-} \otimes \hat{Q}_S + \frac{4}{9}\hat{Q}_S \otimes \hat{Q}_{T_0} + \frac{4}{9}\hat{Q}_{T_0} \otimes \hat{Q}_S + \hat{Q}_{T_+} \otimes \hat{Q}_{T_0} + \hat{Q}_{T_0} \otimes \hat{Q}_{T_+} + \hat{Q}_{T_-} \otimes \hat{Q}_{T_0} + \hat{Q}_{T_0} \otimes \hat{Q}_{T_-}\Big). \quad \text{(S38)}$$

The generic "random fields relaxation" superoperator describes relaxation of the three Cartesian components of the electron spin[10] with relaxation rate, k_{RFR},

$$\hat{\hat{R}}_{\mathrm{RFR}} = k_{\mathrm{RFR}}\Big(\frac{3}{2}\hat{E} \otimes \hat{E} - \hat{S}_{1x} \otimes \hat{S}_{1x}^T - \hat{S}_{1y} \otimes \hat{S}_{1y}^T - \hat{S}_{1z} \otimes \hat{S}_{1z}^T - \hat{S}_{2x} \otimes \hat{S}_{2x}^T - \hat{S}_{2y} \otimes \hat{S}_{2y}^T - \hat{S}_{2z} \otimes \hat{S}_{2z}^T\Big). \quad \text{(S39)}$$

Specific electron spin relaxation terms defining both spin-lattice (T_1) and spin-spin (T_2) relaxation are defined by the following superoperators,[22]

$$\hat{\hat{R}}_{T_1} = k_{T_1}\Big(\hat{E} \otimes \hat{E} - \hat{S}_{1z} \otimes \hat{S}_{1z}^T - \hat{S}_{2z} \otimes \hat{S}_{2z}^T\Big) \quad \text{(S40)}$$

$$\hat{\hat{R}}_{T_2} = k_{T_2}\Big(\hat{E} \otimes \hat{E} - \hat{S}_{1x} \otimes \hat{S}_{1x}^T - \hat{S}_{1y} \otimes \hat{S}_{1y}^T - \hat{S}_{2x} \otimes \hat{S}_{2x}^T - \hat{S}_{2y} \otimes \hat{S}_{2y}^T\Big). \quad \text{(S41)}$$

Under high magnetic field regimes, g-tensor anisotropy plays an important role in radical pair decoherence. This form of relaxation is born from the rotational modulation of the anisotropic electron Zeeman interaction,[23]

$$\hat{\hat{R}}_g = \frac{1}{15}\sum_{j=x,y,z}\Big(\frac{g_{jj} - g_{\mathrm{iso}}}{g_{\mathrm{iso}}}\Big)^2 \omega_0^2 \odot \Big[3J(\omega_0)\Big(\frac{1}{2}\hat{E} \otimes \hat{E} - \hat{S}_{1x} \otimes \hat{S}_{1x}^T - \hat{S}_{1y} \otimes \hat{S}_{1y}^T\Big) + 2J(0)\Big(\frac{1}{2}\hat{E} \otimes \hat{E} - 2\hat{S}_{1z} \otimes \hat{S}_{1z}^T\Big)\Big]. \quad \text{(S42)}$$

Where g_{iso} is the isotropic g-value; g_{jj} denotes the principal components of the g-tensor (g_{xx}, g_{yy}, g_{zz}); ω_0 is the Larmor frequency; $J(\omega)$ is the spectral density,

$$J(\omega) = \frac{\tau_c}{1 + \omega^2\tau_c^2}, \quad \text{(S43)}$$

τ_c is the rotational correlation time.

Incoherent processes in *RadicalPy* Incoherent processes in *RadicalPy* are implemented as a hierarchy of Python classes. Instantiating one of the kinetics or relaxation classes with parameters (usually corresponding to rate constants) yields an object which can be passed to (the constructor of) the simulation object.

S1.2 Semi-classical simulations

A semi-classical method is typically employed when computing radical pair spin dynamics, which involves electron spins hyperfine coupled to a large number of nuclear spins. The reason being is that the size of the spin Hilbert space of coupled electron and nuclear spins increases exponentially with the number of spins. A problem arises when interpreting experimental data involving molecules that typically possess more than 10 nuclear spins, since the required computer memory far exceeds that of commercial desktop computers (see Table S1).[24]

One solution to the exponential scaling problem was first introduced by Schulten and Wolynes in 1978.[25] Their semi-classical approximation describes electron spin precession around a weighted sum of nuclear spin vectors that are fixed in space. Therefore, the number of nuclear spins ceases to matter. Recent extensions of semi-classical theory have been proposed and are beyond the scope of this paper.[26,27] However, we foresee their addition to future versions of *RadicalPy*.

S1.2.1 Schulten-Wolynes theory

Schulten and Wolynes treat the effect of magnetic nuclei as the hyperfine weighted sum of randomly orientated nuclear spin vectors,

$$\mathbf{I}_i = \sum_k a_{ik} \mathbf{I}_{ik}. \tag{S44}$$

Here, a_{ij} represents the hyperfine coupling constant of the kth nuclei. The orientation of $\mathbf{I}_i$ is randomly sampled with the distribution function,

$$f(\mathbf{I}_i) = \left(\frac{\tau_i^2}{4\pi}\right)^{3/2} e^{-I_i^2 \tau_i^2/4}. \tag{S45}$$

Where,

$$\tau_i^2 = \frac{6}{\sum_k a_{ik}^2 I_{ik}(I_{ik}+1)}. \tag{S46}$$

I_{ik} is the nuclear spin quantum number of the kth nuclei interacting with electron spin $\mathbf{S}_i$. The spin Hamiltonian for the Zeeman and hyperfine interactions is given by,

$$\hat{H}_{\mathrm{SW}} = \sum_{i=1,2} \left(g_i \mu_B B_0 \hat{S}_{iz} + \mathbf{I}_i \cdot \hat{\mathbf{S}}_i \right). \tag{S47}$$

Where g_i represent Landé g-factors; μ_B defines the Bohr magneton; B_0 is the external magnetic field; $\hat{S}_i z$ is the electron spin operator in the z-axis.

Schulten-Wolynes theory in *RadicalPy* The Schulten-Wolynes semiclassical approach is implemented as the `semiclassical_mary` function. It uses a `semiclassicalSimulation` object with molecules created using the `all_nuclei` method, which loads all the hyperfine coupling constants from the database required by the Schulten-Wolynes method.

S1.3 Spin Hamiltonian memory allocation

Table S1: Spin Hamiltonian memory allocation comparison for increasing number of spins in Hilbert and Liouville space. The Hamiltonian size used in the semi-classical simulations corresponds to $N = 2$.

**Spins** N	**Hilbert space** $2^N \times 2^N$	**Liouville space** $(2^N)^2 \times (2^N)^2$
2	4 KB	64 KB
3	16 KB	1 MB
4	64 KB	16 MB
5	256 KB	256 MB
6	1 MB	4 GB
7	4 MB	64 GB
8	16 MB	1 TB
9	64 MB	16 TB
10	256 MB	256 TB
11	1 GB	4 PB
12	4 GB	64 PB
13	16 GB	1 EB
14	64 GB	16 EB
15	256 GB	256 EB

Parameters used for simulations

Table S2: Parameters used for simulations on FAD (pH 2.1) depicted in Figures 1, 3, and 4.[29,30]

Parameter	Value	Information
B_0	0 - 30 mT (0.5 mT step size)	External magnetic field
Time	0 - 10 μs (10 ns step size)	Time evolution
Samples	200	Random samples (semi-classical)
HFCs	flavin anion (all), tryptophan cation (all)	*RadicalPy* database
k_{ex}	1.00×10^4 s^{-1}	Groundstate excitation rate
k_{fl}	3.55×10^8 s^{-1}	Fluorescence decay[28]
k_{IC}	1.28×10^9 s^{-1}	Internal conversion
k_{ISC}	3.64×10^8 s^{-1}	Intersystem crossing rate
k_d	3.00×10^5 s^{-1}	Excited triplet decay
k_1	7.00×10^6 s^{-1}	Excited triplet to radical pair
k_{-1}	2.70×10^9 s^{-1}	Radical pair to excited triplet
k_{BET}	1.37×10^7 s^{-1}	Singlet recombination
k_R^T	1.00×10^9 s^{-1}	Triplet excited state relaxation
k_R	7.00×10^7 s^{-1}	Random fields relaxation
k_{STD}	7.00×10^8 s^{-1}	Singlet-triplet dephasing

Table S3: Parameters used for simulations on FAD-Trp encapsulated inside an AOT reverse micelle displayed in Figure 5.

Parameters	Value	Information
B_0	0 - 30 mT (0.1 mT step size)	External magnetic field
Time	0 - 10 μs (10 ns step size)	Time evolution
Samples	100	Random samples (semi-classical)
HFCs	flavin anion (all), tryptophan cation (all)	*RadicalPy* database
k_{BET}	8.00×10^6 s^{-1}	Singlet recombination[31]
k_Q	5.00×10^6 s^{-1}	Excited triplet quenching rate[31]
k_{FR}	5.00×10^5 s^{-1}	Free radical escape rate[31]
k_{STD}	1.17×10^7 s^{-1}	ST-dephasing (MD simulation)

Table S4: Parameters used for simulations on FAD-Trp or FAD-Z cryptochrome anisotropies shown in Figure 6.

**Parameter**	**Value**	**Information**
B_0	50 µT	External magnetic field
Time	0 – 15 µs (5 ns step size)	Time evolution
θ	0 - π (35 steps)	Rotation angle θ
ϕ	0 - 2π (58 steps)	Rotation angle ϕ
HFC	$\begin{pmatrix} 0.280 & -0.138 & 0.678 \\ -0.138 & 0.043 & -0.331 \\ 0.678 & -0.331 & 1.412 \end{pmatrix}$	FAD (N_5)
HFC	$\begin{pmatrix} 0.944 & -0.019 & 0.030 \\ -0.019 & 1.091 & -0.065 \\ 0.030 & -0.065 & 1.070 \end{pmatrix}$	Trp (H_β)
D	$\begin{pmatrix} 0.174 & 0.000 & 0.000 \\ 0.000 & 0.174 & 0.000 \\ 0.000 & 0.000 & 0.349 \end{pmatrix}$	Anisotropic dipolar coupling (FAD-Z)
D	$\begin{pmatrix} -0.225 & 0.156 & 0.198 \\ 0.156 & 0.117 & -0.082 \\ 0.198 & -0.082 & 0.107 \end{pmatrix}$	Anisotropic dipolar coupling (FAD-Trp)
k	1.00×10^6 s^{-1}	Exponential model

Table S5: Parameters used for simulations on anthracene shown in Figure 7.

**Parameters**	**Value**	**Information**
B_0	$0 - 2.5$ T (10 mT step size)	External magnetic field
θ	$\pi/4$	Rotation angle θ
ϕ	0	Rotation angle ϕ
D	-6.2 mT	Zero-field splitting
E	35 mT	Zero-field splitting
J	499.55 mT	Exchange interaction
k_{SF}	1.00	Singlet fission ratio
k_{TTA}	1.10×10^9 $\mathrm{M^{-1}s^{-1}}$	Triplet-triplet annihilation
k_{Diss}	2.80×10^9 $\mathrm{s^{-1}}$	Triplet dissociation

S1.4 Code listings

Figures 1, 3, and 4

```python
#! /usr/bin/env python

from pathlib import Path

import matplotlib.pyplot as plt
import numpy as np

import radicalpy as rp
from radicalpy.classical import Rate, RateEquations, latex_eqlist_to_align, latexify
from radicalpy.experiments import kine_quantum_mary
from radicalpy.plot import plot_3d_results, plot_bhalf_time, plot_general
from radicalpy.relaxation import RandomFields, SingletTripletDephasing
from radicalpy.simulation import Basis, Molecule, SemiclassicalSimulation
from radicalpy.utils import Bhalf_fit, is_fast_run


def main(Bmax=30, dB=0.5, tmax=10e-6, dt=10e-9):

    # Parameters
    time = np.arange(0, tmax, dt)
    Bs = np.arange(0, Bmax, dB)
    num_samples = 200
    scale_factor = 1  # 4e-1
    kr = 7e7  # 1.7e6  # radical pair relaxation rate
    kstd = 7e8  # 5e9  # spin dephasing rate
    # relaxation = RandomFields(kr)  # relaxation model
    # relaxation = SingletTripletDephasing(kr)  # relaxation model

    # Axes for orientation of 3D plots
```

```python
# azim = -135
# dist = 10
# elev = 35

# Load reference spectra
path = Path("./examples/data/fad_kinetics")
radical_spectrum = 1e3 * np.genfromtxt(path / "fad_radical_spectrum.txt")
triplet_spectrum = 1e3 * np.genfromtxt(path / "fad_triplet_spectrum.txt")
wavelength = np.genfromtxt(path / "fad_radical_wavelength.txt")
groundstate_spectrum = np.genfromtxt(path / "fad_groundstate_spectrum.txt")
groundstate_wavelength = np.genfromtxt(
    path / "fad_groundstate_spectrum_wavelength.txt"
)
emission_spectrum = np.genfromtxt(path / "fad_emission_spectrum.txt")
emission_wavelength = np.genfromtxt(path / "fad_emission_spectrum_wavelength.txt")

flavin = Molecule.all_nuclei("fad")
adenine = Molecule.all_nuclei("fad")
sim = SemiclassicalSimulation([flavin, adenine], basis=Basis.ZEEMAN)
bhalf = rp.estimations.Bhalf_theoretical_hyperfine(sim)
khfc_new = rp.estimations.k_ST_mixing(bhalf)
khfc = 8e7  # spin-state mixing rate
khfc_ratio = khfc_new / khfc

# Kinetic simulation of FAD at pH 2.1.

# FAD kinetic parameters
kex = Rate(1e4, "k_{ex}")  # groundstate excitation rate
kfl = Rate(3.55e8, "k_{fl}")  # fluorescence rate
kic = Rate(1.28e9, "k_{IC}")  # internal conversion rate
kisc = Rate(3.64e8, "k_{ISC}")  # intersystem crossing rate
kd = Rate(7e6, "k_d")  # protonated triplet to ground state
k1 = Rate(7e6, "k_1")  # protonated triplet to RP
```

```
km1 = Rate(2.7e9 * khfc_ratio, "k_{-1}")  # RP to protonated triplet
krt = Rate(1e9, "k^R_T")  # triplet state relaxation rate
kbet = Rate(1.1e7 * khfc_ratio, "k_{BET}")  # singlet recombination rate
pH = 2.1  # pH of the solution
Hp = Rate(10**-pH, "H^+")  # concentration of hydrogen ions

# Rate equations
S0, S1, T1p, T10, T1m = "S_0", "S_1", "T_1^+", "T_1^0", "T_1^-"
SS, STp, ST0, STm = "SS", "ST_+", "ST_0", "ST_-"
TpS, TpTp, TpT0, TpTm = "T_+S", "T_+T_+", "T_+T_0", "T_+T_-"
T0S, T0Tp, T0T0, T0Tm = "T_0S", "T_0T_+", "T_0T_0", "T_0T_-"
TmS, TmTp, TmT0, TmTm = "T_-S", "T_-T_+", "T_-T_0", "T_-T_-"

base = {}
base[S0] = {
    S0: -kex,
    S1: kfl + kic,
    T1p: kd,
    T10: kd,
    T1m: kd,
    SS: kbet,
}
base[S1] = {
    S0: kex,
    S1: -(kfl + kic + 3 * kisc),
}
base[T1p] = {
    S1: kisc,
    T1p: -(kd + k1 + krt),
    T10: krt,
    TpTp: km1 * Hp,
}
base[T10] = {
```

```
    S1: kisc,
    T1p: krt,
    T10: -(kd + k1 + 2 * krt),
    T1m: krt,
    T0T0: km1 * Hp,
}
base[T1m] = {
    S1: kisc,
    T10: krt,
    T1m: -(kd + k1 + krt),
    TmTm: km1 * Hp,
}

base[SS] = {
    SS: -(kbet),
}
base[STp] = {
    STp: -(kbet + km1 * Hp) / 2,
}
base[ST0] = {
    ST0: -(kbet + km1 * Hp) / 2,
}
base[STm] = {
    STm: -(kbet + km1 * Hp) / 2,
}

base[TpS] = {
    TpS: -(kbet + km1 * Hp) / 2,
}
base[TpTp] = {
    T1p: k1,
    TpTp: -(km1 * Hp),
}
```

```
base[TpT0] = {
    TpT0: -(km1 * Hp),
}
base[TpTm] = {
    TpTm: -(km1 * Hp),
}

base[T0S] = {
    T0S: -(kbet + km1 * Hp) / 2,
}
base[T0Tp] = {
    T0Tp: -(km1 * Hp),
}
base[T0T0] = {
    T10: k1,
    T0T0: -(km1 * Hp),
}
base[T0Tm] = {
    T0Tm: -(km1 * Hp),
}

base[TmS] = {
    TmS: -(kbet + km1 * Hp) / 2,
}
base[TmTp] = {
    TmTp: -(km1 * Hp),
}
base[TmT0] = {
    TmT0: -(km1 * Hp),
}
base[TmTm] = {
    T1m: k1,
    TmTm: -(km1 * Hp),
```

```
}

rate_eq = RateEquations(base)
mat = rate_eq.matrix.todense()
rho0 = np.array(
    [
        0,  # S0
        0,  # S1
        1 / 3,  # T1+
        1 / 3,  # T10
        1 / 3,  # T1-
        0,  # SS
        0,  # ST+
        0,  # ST0
        0,  # ST-
        0,  # T+S
        0,  # T+T+
        0,  # T+T0
        0,  # T+T-
        0,  # T0S
        0,  # T0T+
        0,  # T0T0
        0,  # T0T-
        0,  # T-S
        0,  # T-T+
        0,  # T-0
        0,  # T-T-
    ]
)

latex_equations = latex_eqlist_to_align(latexify(base))
# print(latex_equations)
```

```python
results = kine_quantum_mary(
    sim,
    num_samples,
    rho0,
    radical_pair=[5, 21],
    ts=time,
    Bs=Bs,
    D=0,
    J=0,
    kinetics=mat,
    relaxations=[RandomFields(kr), SingletTripletDephasing(kstd)],
)

# np.save("./examples/data/fad_mary/results_new.npy", results)

total_yield = np.zeros((len(time), len(Bs), len(wavelength)), dtype=complex)
zero_field = np.zeros((len(time), len(Bs), len(wavelength)), dtype=complex)
mary = np.zeros((len(time), len(Bs), len(wavelength)), dtype=complex)
total_yield_groundstate = np.zeros(
    (len(time), len(Bs), len(groundstate_wavelength)), dtype=complex
)
zero_field_groundstate = np.zeros(
    (len(time), len(Bs), len(groundstate_wavelength)), dtype=complex
)
mary_groundstate = np.zeros(
    (len(time), len(Bs), len(groundstate_wavelength)), dtype=complex
)
total_yield_emission = np.zeros(
    (len(time), len(Bs), len(emission_wavelength)), dtype=complex
)
zero_field_emission = np.zeros(
    (len(time), len(Bs), len(emission_wavelength)), dtype=complex
)
```

```python
mary_emission = np.zeros(
    (len(time), len(Bs), len(emission_wavelength)), dtype=complex
)

radical_pair_yield = (
    results["yield"][:, 5, :]
    + results["yield"][:, 10, :]
    + results["yield"][:, 15, :]
    + results["yield"][:, 20, :]
)
triplet_yield = (
    results["yield"][:, 2, :]
    + results["yield"][:, 3, :]
    + results["yield"][:, 4, :]
)
groundstate_yield = results["yield"][:, 0, :]

for i, r in enumerate(radical_spectrum):
    for j, t in enumerate(triplet_spectrum):
        total_yield[:, :, j + 1] = (
            (r * radical_pair_yield) + (t * (2 * triplet_yield))
        ) * scale_factor

for i in range(0, len(wavelength)):
    for j in range(0, len(Bs)):
        zero_field[:, j, i] = total_yield[:, 0, i]

for i, g in enumerate(groundstate_spectrum):
    total_yield_groundstate[:, :, i] = (g * groundstate_yield) * (scale_factor)

for i in range(0, len(groundstate_wavelength)):
    for j in range(0, len(Bs)):
        zero_field_groundstate[:, j, i] = total_yield_groundstate[:, 0, i]
```

```
for i, g in enumerate(emission_spectrum):
    total_yield_emission[:, :, i] = (g * groundstate_yield) * 100

for i in range(0, len(emission_wavelength)):
    for j in range(0, len(Bs)):
        zero_field_emission[:, j, i] = total_yield_emission[:, 0, i]

mary = np.real(total_yield - zero_field)
mary_groundstate = np.real(total_yield_groundstate - zero_field_groundstate)
mary_emission = np.real(total_yield_emission - zero_field_emission)

# np.savetxt(
#     "./examples/data/fad_kinetics/semiclassical_kinetics_new3.txt",
#     mary[:, 1],
# )
# np.savetxt("./examples/data/fad_kinetics/semiclassical_kinetics_time3.txt", time)

mfe_max = np.zeros(len(wavelength), dtype=complex)
for i in range(1, len(wavelength)):
    mfe_max[i] = mary[:, -1, i].max()

mfe_groundstate_max = np.zeros(len(groundstate_wavelength), dtype=complex)
for i in range(1, len(groundstate_wavelength)):
    mfe_groundstate_max[i] = mary_groundstate[:, -1, i].min()

mfe_emission_max = np.zeros(len(emission_wavelength), dtype=complex)
for i in range(1, len(emission_wavelength)):
    mfe_emission_max[i] = mary_emission[:, -1, i].min()

xlabel = r"Wavelength / nm"
ylabel = r"$\Delta \Delta A$"
plot_general(
```

```
    groundstate_wavelength[1:],
    np.real(mfe_groundstate_max)[1:],
    xlabel,
    ylabel,
    "r-",
    label=f"{Bs.max(): .0f} mT",
)
path = __file__[:-3] + f"_{0}.png"
plt.savefig(path, dpi=300, bbox_inches="tight")
plt.close()

xlabel = r"Wavelength / nm"
ylabel = r"$\Delta I_F$"
plot_general(
    emission_wavelength[1:],
    np.real(mfe_emission_max)[1:],
    xlabel,
    ylabel,
    "r-",
    label=f"{Bs.max(): .0f} mT",
)
path = __file__[:-3] + f"_{1}.png"
plt.savefig(path, dpi=300, bbox_inches="tight")
plt.close()

xlabel = r"Wavelength / nm"
ylabel = r"$\Delta \Delta A$"
plot_general(
    wavelength,
    np.real(mfe_max),
    xlabel,
    ylabel,
    "r-",
```

```
    label=f"{Bs.max(): .0f} mT",
)
path = __file__[:-3] + f"_{2}.png"
plt.savefig(path, dpi=300, bbox_inches="tight")
plt.close()

n = 100
f = 25
wl = -1
factor = 1e6
colors_time = plt.colormaps.get_cmap("viridis").resampled(len(time)).colors
colors_field = plt.colormaps.get_cmap("viridis").resampled(len(Bs)).colors

xlabel = r"Wavelength / nm"
ylabel = r"$\Delta \Delta A$"
for i in range(0, len(time), n):
    plot_general(
        groundstate_wavelength,
        mary_groundstate[i, -1, :],
        xlabel,
        ylabel,
        style="-",
        label=f"{time[i] * factor: .0f} $\\mu s$",
        colors=colors_time[i],
    )
path = __file__[:-3] + f"_{3}.png"
plt.savefig(path, dpi=300, bbox_inches="tight")
plt.close()

xlabel = r"$B_0$ / mT"
ylabel = r"$\Delta \Delta A$"
for i in range(0, len(time), n):
    plot_general(
```

```
        Bs,
        mary_groundstate[i, :, wl],
        xlabel,
        ylabel,
        style="-",
        label=f"{time[i] * factor: .0f} $\\mu s$",
        colors=colors_time[i],
    )
path = __file__[:-3] + f"_{4}.png"
plt.savefig(path, dpi=300, bbox_inches="tight")
plt.close()

xlabel = r"Time / $\mu s$"
ylabel = r"$\Delta \Delta A$"
for i in range(0, len(Bs), f):
    plot_general(
        time,
        mary_groundstate[:, i, wl],
        xlabel,
        ylabel,
        style="-",
        label=f"{Bs[i]: .1f} mT",
        colors=colors_field[i],
        factor=1e6,
    )
path = __file__[:-3] + f"_{5}.png"
plt.savefig(path, dpi=300, bbox_inches="tight")
plt.close()

xlabel = r"Wavelength / nm"
ylabel = r"$\Delta I_F$"
for i in range(0, len(time), n):
    plot_general(
```

```
        emission_wavelength,
        mary_emission[i, -1, :],
        xlabel,
        ylabel,
        style="-",
        label=f"{time[i] * factor: .0f} $\\mu s$",
        colors=colors_time[i],
    )
path = __file__[:-3] + f"_{6}.png"
plt.savefig(path, dpi=300, bbox_inches="tight")
plt.close()

xlabel = r"$B_0$ / mT"
ylabel = r"$\Delta I_F$"
for i in range(0, len(time), n):
    plot_general(
        Bs,
        mary_emission[i, :, wl],
        xlabel,
        ylabel,
        style="-",
        label=f"{time[i] * factor: .0f} $\\mu s$",
        colors=colors_time[i],
    )
path = __file__[:-3] + f"_{7}.png"
plt.savefig(path, dpi=300, bbox_inches="tight")
plt.close()

xlabel = r"Time / $\mu s$"
ylabel = r"$\Delta I_F$"
for i in range(0, len(Bs), f):
    plot_general(
        time,
```

```
        mary_emission[:, i, wl],
        xlabel,
        ylabel,
        style="-",
        label=f"{Bs[i]: .1f} mT",
        colors=colors_field[i],
        factor=1e6,
    )
path = __file__[:-3] + f"_{8}.png"
plt.savefig(path, dpi=300, bbox_inches="tight")
plt.close()

xlabel = r"Wavelength / nm"
ylabel = r"$\Delta \Delta A$"
for i in range(0, len(time), n):
    plot_general(
        wavelength,
        mary[i, -1, :],
        xlabel,
        ylabel,
        style="-",
        label=f"{time[i] * factor: .0f} $\\mu s$",
        colors=colors_time[i],
    )
path = __file__[:-3] + f"_{9}.png"
plt.savefig(path, dpi=300, bbox_inches="tight")
plt.close()

xlabel = r"$B_0$ / mT"
ylabel = r"$\Delta \Delta A$"
for i in range(0, len(time), n):
    plot_general(
        Bs,
```

```
            mary[i, :, wl],
            xlabel,
            ylabel,
            style="-",
            label=f"{time[i] * factor: .0f} $\\mu s$",
            colors=colors_time[i],
        )
    path = __file__[:-3] + f"_{10}.png"
    plt.savefig(path, dpi=300, bbox_inches="tight")
    plt.close()

    xlabel = r"Time / $\mu s$"
    ylabel = r"$\Delta \Delta A$"
    for i in range(0, len(Bs), f):
        plot_general(
            time,
            mary[:, i, wl],
            xlabel,
            ylabel,
            style="-",
            label=f"{Bs[i]: .1f} mT",
            colors=colors_field[i],
            factor=1e6,
        )
    path = __file__[:-3] + f"_{11}.png"
    plt.savefig(path, dpi=300, bbox_inches="tight")
    plt.close()
    xlabel = r"Wavelength / nm"
    ylabel = r"$\Delta \Delta A$"
    for i in range(0, len(time), n):
        plot_general(
            groundstate_wavelength,
            mary_groundstate[i, -1, :],
```

```
        xlabel,
        ylabel,
        style="-",
        label=f"{time[i] * factor: .0f} $\\mu s$",
    )
path = __file__[:-3] + f"_{3}.png"
plt.savefig(path, dpi=300, bbox_inches="tight")
plt.close()

xlabel = r"$B_0$ / mT"
ylabel = r"$\Delta \Delta A$"
for i in range(0, len(time), n):
    plot_general(
        Bs,
        mary_groundstate[i, :, wl],
        xlabel,
        ylabel,
        style="-",
        label=f"{time[i] * factor: .0f} $\\mu s$",
    )
path = __file__[:-3] + f"_{4}.png"
plt.savefig(path, dpi=300, bbox_inches="tight")
plt.close()

xlabel = r"Time / $\mu s$"
ylabel = r"$\Delta \Delta A$"
for i in range(0, len(Bs), f):
    plot_general(
        time,
        mary_groundstate[:, i, wl],
        xlabel,
        ylabel,
        style="-",
```

```
        label=f"{Bs[i]: .1f} mT",
        factor=1e6,
    )
path = __file__[:-3] + f"_{5}.png"
plt.savefig(path, dpi=300, bbox_inches="tight")
plt.close()

xlabel = r"Wavelength / nm"
ylabel = r"$\Delta I_F$"
for i in range(0, len(time), n):
    plot_general(
        emission_wavelength,
        mary_emission[i, -1, :],
        xlabel,
        ylabel,
        style="-",
        label=f"{time[i] * factor: .0f} $\\mu s$",
    )
path = __file__[:-3] + f"_{6}.png"
plt.savefig(path, dpi=300, bbox_inches="tight")
plt.close()

xlabel = r"$B_0$ / mT"
ylabel = r"$\Delta I_F$"
for i in range(0, len(time), n):
    plot_general(
        Bs,
        mary_emission[i, :, wl],
        xlabel,
        ylabel,
        style="-",
        label=f"{time[i] * factor: .0f} $\\mu s$",
    )
```

```
path = __file__[:-3] + f"_{7}.png"
plt.savefig(path, dpi=300, bbox_inches="tight")
plt.close()

xlabel = r"Time / $\mu s$"
ylabel = r"$\Delta I_F$"
for i in range(0, len(Bs), f):
    plot_general(
        time,
        mary_emission[:, i, wl],
        xlabel,
        ylabel,
        style="-",
        label=f"{Bs[i]: .1f} mT",
        factor=1e6,
    )
path = __file__[:-3] + f"_{8}.png"
plt.savefig(path, dpi=300, bbox_inches="tight")
plt.close()

xlabel = r"Wavelength / nm"
ylabel = r"$\Delta \Delta A$"
for i in range(0, len(time), n):
    plot_general(
        wavelength,
        mary[i, -1, :],
        xlabel,
        ylabel,
        style="-",
        label=f"{time[i] * factor: .0f} $\\mu s$",
    )
path = __file__[:-3] + f"_{9}.png"
plt.savefig(path, dpi=300, bbox_inches="tight")
```

```
plt.close()

xlabel = r"$B_0$ / mT"
ylabel = r"$\Delta \Delta A$"
for i in range(0, len(time), n):
    plot_general(
        Bs,
        mary[i, :, wl],
        xlabel,
        ylabel,
        style="-",
        label=f"{time[i] * factor: .0f} $\\mu s$",
    )
path = __file__[:-3] + f"_{10}.png"
plt.savefig(path, dpi=300, bbox_inches="tight")
plt.close()

xlabel = r"Time / $\mu s$"
ylabel = r"$\Delta \Delta A$"
for i in range(0, len(Bs), f):
    plot_general(
        time,
        mary[:, i, wl],
        xlabel,
        ylabel,
        style="-",
        label=f"{Bs[i]: .1f} mT",
        factor=1e6,
    )
path = __file__[:-3] + f"_{11}.png"
plt.savefig(path, dpi=300, bbox_inches="tight")
plt.close()
```

```python
# Calculate time evolution of the B1/2
bhalf_time = np.zeros((len(mary_groundstate)))
fit_time = np.zeros((len(Bs), len(mary_groundstate)))
fit_error_time = np.zeros((2, len(mary_groundstate)))
R2_time = np.zeros((len(mary_groundstate)))

for i in range(2, len(mary_groundstate)):
    (
        bhalf_time[i],
        fit_time[:, i],
        fit_error_time[:, i],
        R2_time[i],
    ) = Bhalf_fit(Bs, mary_groundstate[i, :, wl])

plot_bhalf_time(
    time[2:], bhalf_time[2:], fit_error_time[:, 2:] / np.sqrt(num_samples)
)
path = __file__[:-3] + f"_{18}.png"
plt.savefig(path, dpi=300, bbox_inches="tight")
plt.close()

bhalf_time = np.zeros((len(mary_emission)))
fit_time = np.zeros((len(Bs), len(mary_emission)))
fit_error_time = np.zeros((2, len(mary_emission)))
R2_time = np.zeros((len(mary_emission)))

for i in range(2, len(mary_emission)):
    (
        bhalf_time[i],
        fit_time[:, i],
        fit_error_time[:, i],
        R2_time[i],
    ) = Bhalf_fit(Bs, mary_emission[i, :, wl])
```

```python
plot_bhalf_time(time, bhalf_time, fit_error_time / np.sqrt(num_samples))
path = __file__[:-3] + f"_{19}.png"
plt.savefig(path, dpi=300, bbox_inches="tight")
plt.close()

bhalf_time = np.zeros((len(mary)))
fit_time = np.zeros((len(Bs), len(mary)))
fit_error_time = np.zeros((2, len(mary)))
R2_time = np.zeros((len(mary)))

for i in range(2, len(mary)):
    (
        bhalf_time[i],
        fit_time[:, i],
        fit_error_time[:, i],
        R2_time[i],
    ) = Bhalf_fit(Bs, mary[i, :, wl])

plot_bhalf_time(time, bhalf_time, fit_error_time / np.sqrt(num_samples))
path = __file__[:-3] + f"_{20}.png"
plt.savefig(path, dpi=300, bbox_inches="tight")
plt.close()

# 3D plots
xlabel = r"Wavelength / nm"
ylabel = r"Time / $\mu s$"
zlabel = r"$\Delta \Delta A$"
plot_3d_results(
    groundstate_wavelength,
    results["ts"],
    mary_groundstate[:, -1, :],
    xlabel,
```

```
    ylabel,
    zlabel,
)
path = __file__[:-3] + f"_{12}.png"
plt.savefig(path, dpi=300)
plt.close()

xlabel = r"Wavelength / nm"
ylabel = r"$B_0$ / mT"
zlabel = r"$\Delta \Delta A$"
plot_3d_results(
    groundstate_wavelength,
    results["Bs"],
    mary_groundstate[len(mary_groundstate) // 4, :, :],
    xlabel,
    ylabel,
    zlabel,
    factor=1,
)
path = __file__[:-3] + f"_{13}.png"
plt.savefig(path, dpi=300)
plt.close()

xlabel = r"Wavelength / nm"
ylabel = r"Time / $\mu s$"
zlabel = r"$\Delta I_F$"
plot_3d_results(
    emission_wavelength,
    results["ts"],
    mary_emission[:, -1, :],
    xlabel,
    ylabel,
    zlabel,
```

```
)
path = __file__[:-3] + f"_{14}.png"
plt.savefig(path, dpi=300)
plt.close()

xlabel = r"Wavelength / nm"
ylabel = r"$B_0$ / mT"
zlabel = r"$\Delta I_F$"
plot_3d_results(
    emission_wavelength,
    results["Bs"],
    mary_emission[len(mary_emission) // 4, :, :],
    xlabel,
    ylabel,
    zlabel,
    factor=1,
)
path = __file__[:-3] + f"_{15}.png"
plt.savefig(path, dpi=300)
plt.close()

xlabel = r"Wavelength / nm"
ylabel = r"Time / $\mu s$"
zlabel = r"$\Delta \Delta A$"
plot_3d_results(
    wavelength,
    results["ts"],
    mary[:, -1, :],
    xlabel,
    ylabel,
    zlabel,
)
path = __file__[:-3] + f"_{16}.png"
```

```python
    plt.savefig(path, dpi=300)
    plt.close()

    xlabel = r"Wavelength / nm"
    ylabel = r"$B_0$ / mT"
    zlabel = r"$\Delta \Delta A$"
    plot_3d_results(
        wavelength,
        results["Bs"],
        mary[len(mary) // 4, :, :],
        xlabel,
        ylabel,
        zlabel,
        factor=1,
    )
    path = __file__[:-3] + f"_{17}.png"
    plt.savefig(path, dpi=300)
    plt.close()


if __name__ == "__main__":
    if is_fast_run():
        main(Bmax=10, dB=2, tmax=10e-6, dt=1e-6)
    else:
        main()
```

Figure 5

```python
#! /usr/bin/env python

import matplotlib.pyplot as plt
import numpy as np

from radicalpy.data import Molecule
from radicalpy.estimations import (
    autocorrelation_fit,
    exchange_interaction_in_solution_MC,
    k_STD,
)
from radicalpy.experiments import semiclassical_mary
from radicalpy.kinetics import Haberkorn
from radicalpy.plot import (
    plot_3d_results,
    plot_autocorrelation_fit,
    plot_bhalf_time,
    plot_exchange_interaction_in_solution,
)
from radicalpy.relaxation import SingletTripletDephasing
from radicalpy.simulation import SemiclassicalSimulation, State
from radicalpy.utils import (
    Bhalf_fit,
    autocorrelation,
    is_fast_run,
    read_trajectory_files,
)


def main(
    ts=np.arange(0, 10e-6, 10e-9),
```

```python
    Bs=np.arange(0, 30, 0.1),
    num_samples=100,
):
    flavin = Molecule.all_nuclei("flavin_anion")
    trp = Molecule.all_nuclei("tryptophan_cation")
    sim = SemiclassicalSimulation([flavin, trp])

    trajectory_data = read_trajectory_files(
        "./examples/data/md_fad_trp_aot", scale=1e-10
    )
    trajectory_ts = (
        np.linspace(0, len(trajectory_data), len(trajectory_data)) * 5e-12 * 1e9
    )
    j = exchange_interaction_in_solution_MC(trajectory_data[:, 1], J0=5)

    plot_exchange_interaction_in_solution(trajectory_ts, trajectory_data, j)
    path = __file__[:-3] + f"_{0}.png"
    plt.savefig(path, dpi=300, bbox_inches="tight")
    plt.close()

    acf_j = autocorrelation(j, factor=1)
    zero_point_crossing_j = np.where(np.diff(np.sign(acf_j)))[0][0]
    t_j_max = max(trajectory_ts[:zero_point_crossing_j]) * 1e-9
    t_j = np.linspace(5e-12, t_j_max, zero_point_crossing_j)

    acf_j_fit = autocorrelation_fit(t_j, j, 5e-12, t_j_max)
    plot_autocorrelation_fit(t_j, acf_j, acf_j_fit, zero_point_crossing_j)
    path = __file__[:-3] + f"_{1}.png"
    plt.savefig(path, dpi=300, bbox_inches="tight")
    plt.close()

    kstd = k_STD(-j, acf_j_fit["tau_c"])
    # kstd = 11681368.059456564
```

```
triplet_excited_state_quenching_rate = 5e6
recombination_rate = 8e6
free_radical_escape_rate = 5e5

results = semiclassical_mary(
    sim=sim,
    num_samples=num_samples,
    init_state=State.TRIPLET,
    ts=ts,
    Bs=Bs,
    D=0,
    J=0,
    triplet_excited_state_quenching_rate=triplet_excited_state_quenching_rate,
    free_radical_escape_rate=free_radical_escape_rate,
    kinetics=[Haberkorn(recombination_rate, State.SINGLET)],
    relaxations=[SingletTripletDephasing(kstd)],
    scale_factor=0.005,
)

# Calculate time evolution of the B1/2
bhalf_time = np.zeros((len(results["MARY"])))
fit_time = np.zeros((len(Bs), len(results["MARY"])))
fit_error_time = np.zeros((2, len(results["MARY"])))
R2_time = np.zeros((len(results["MARY"])))

for i in range(2, len(results["MARY"])):
    (
        bhalf_time[i],
        fit_time[:, i],
        fit_error_time[:, i],
        R2_time[i],
    ) = Bhalf_fit(Bs, results["MARY"][i, :])
```

```python
    plot_bhalf_time(ts, bhalf_time, fit_error_time)
    path = __file__[:-3] + f"_{2}.png"
    plt.savefig(path, dpi=300, bbox_inches="tight")
    plt.close()

    xlabel = "$B_0$ / mT"
    ylabel = "Time / $\mu s$"
    zlabel = "$\Delta \Delta A$"
    plot_3d_results(
        xdata=Bs,
        ydata=ts,
        zdata=results["MARY"],
        xlabel=xlabel,
        ylabel=ylabel,
        zlabel=zlabel,
        factor=1e6,
    )
    path = __file__[:-3] + f"_{3}.png"
    plt.savefig(path, dpi=300)
    plt.close()


if __name__ == "__main__":
    if is_fast_run():
        main(num_samples=4)
    else:
        main()
```

Figure 6

```python
#! /usr/bin/env python

import matplotlib.pyplot as plt
import numpy as np

import radicalpy as rp
from radicalpy import kinetics, relaxation
from radicalpy.experiments import anisotropy
from radicalpy.simulation import State
from radicalpy.utils import is_fast_run


def main(theta_steps=35, phi_steps=58, tmax=15e-6, dt=5e-9):
    fad_n5_hfc = np.array(
        [
            [0.280, -0.138, 0.678],
            [-0.138, 0.043, -0.331],
            [0.678, -0.331, 1.412],
        ]
    )

    trp_hbeta_hfc = np.array(
        [
            [0.944, -0.019, 0.030],
            [-0.019, 1.091, -0.065],
            [0.030, -0.065, 1.070],
        ]
    )

    dipolar = (
        np.array(
```

```
            [
                [-0.225, 0.156, 0.198],
                [0.156, 0.117, -0.082],
                [0.198, -0.082, 0.107],
            ]
        )
        * rp.data.Isotope("E").gamma_mT
    )

    theta = np.linspace(0, np.pi, theta_steps)
    phi = np.linspace(0, 2 * np.pi, phi_steps)

    flavin = rp.simulation.Molecule.fromisotopes(isotopes=["14N"], hfcs=[fad_n5_hfc])
    trp = rp.simulation.Molecule.fromisotopes(isotopes=["1H"], hfcs=[trp_hbeta_hfc])
    sim = rp.simulation.HilbertSimulation([flavin, trp])

    time = np.arange(0, tmax, dt)
    B0 = 0.05
    k = 1e6

    results = anisotropy(
        sim,
        init_state=State.SINGLET,
        obs_state=State.SINGLET,
        time=time,
        theta=theta,
        phi=phi,
        B0=B0,
        D=dipolar,
        J=0,
        kinetics=[kinetics.Exponential(k)],
    )
```

```python
    Y = results["product_yield_sums"]
    delta_phi_s, gamma_s = rp.utils.yield_anisotropy(Y, theta, phi)
    Y_av = rp.utils.spherical_average(Y, theta, phi)
    Y = Y - Y_av

    np.save("Y", Y)
    np.save("theta", theta)
    np.save("phi", phi)
    rp.plot.anisotropy_surface(theta, phi, Y)

    print(f"{Y_av=}")
    print(f"{delta_phi_s=}")
    print(f"{gamma_s=}")
    # plt.show()
    path = __file__[:-3] + f"_{3}.png"
    plt.savefig(path)

    return 0


if __name__ == "__main__":
    if is_fast_run():
        main(theta_steps=7, phi_steps=6, tmax=10e-6, dt=1e-6)
    else:
        main()
```

Figure 7

```python
#! /usr/bin/env python

import matplotlib.pyplot as plt
import numpy as np
from radicalpy.data import Triplet
from radicalpy.experiments import steady_state_mary
from radicalpy.kinetics import Haberkorn, HaberkornFree
from radicalpy.simulation import Basis, LiouvilleSimulation, State


def main(
    Bs=np.arange(0, 2500, 10),
    D=-6.2,
    E=35,
    J=499.55,
    ksf=1,
    ktta=1.1e9,
    kdiss=2.8e9,
):
    m = Triplet()
    sim = LiouvilleSimulation(molecules=[m, m], basis=Basis.ZEEMAN)
    rhos, Phi_s = steady_state_mary(
        sim,
        obs=State.TP_SINGLET,
        Bs=Bs,
        D=D,
        E=E,
        J=J,
        theta=np.pi / 4,
        phi=0,
        kinetics=[Haberkorn(ktta, State.TP_SINGLET), HaberkornFree(kdiss)],
```

```
)
rhos *= ksf
Phi_s *= ksf * ktta

MFE = ((np.abs(Phi_s) - np.abs(Phi_s[0])) / np.abs(Phi_s[0])) * 100

E = np.zeros([len(Bs), len(rhos[0])], dtype=np.complex_)

for i, B0 in enumerate(Bs):
    H = sim.total_hamiltonian(B0=B0, D=D, J=J)
    eigval = np.linalg.eigh(H)
    E[i] = eigval[0]  # 0 = eigenvalues, 1 = eigenvectors

# Plotting
fig = plt.figure()
gs = fig.add_gridspec(2, hspace=0)
axs = gs.subplots(sharex=True)
axs[0].set_facecolor("none")
axs[1].set_facecolor("none")
axs[0].grid(False)
plt.axis("on")
plt.rc("axes", edgecolor="k")
axs[0].plot(Bs / J, MFE, linewidth=3, color="tab:red")
axs[1].plot(Bs / J, np.real(E[:, ::-1]) / J, linewidth=3)
axs[1].axvline(x=1.5, ymax=0.4, color="k", linestyle="--")
axs[1].axvline(x=3, ymax=0.4, color="k", linestyle="--")
plt.xscale("linear")
axs[1].set_xlabel("$g \mu_B B_0$ / J", size=18)
axs[0].set_ylabel("MFE (%)", size=18)
axs[1].set_ylabel("E / J", size=18)
axs[0].tick_params(labelsize=14)
axs[1].tick_params(labelsize=14)
plt.ylim(-10, 11)
```

```
    fig.set_size_inches(10, 10)
    plt.show()


if __name__ == "__main__":
    main()
```

Figure S1

```python
#! /usr/bin/env python


from pathlib import Path
import dot2tex
import matplotlib.pyplot as plt
import numpy as np
from radicalpy.classical import Rate, RateEquations, latex_eqlist_to_align, latexify



def main():
    # Simple example of a RP.

    # geminate RP to free radical separation
    # ST-mixing rate
    # RP relaxation rate
    # reverse electron transfer of RP to groundstate

    # kinetic parameters
    ke = Rate(1e6, "k_{E}")
    kst = Rate(8e7, "k_{ST}")
    krlx = Rate(2e6, "k_{Rlx}")
    kr = Rate(1e8, "k_{R}")

    # Rate equations
    S, Tp, T0, Tm = "S", "T_+", "T_0", "T_-"
    off = {}
    off[S] = {S: -(3 * kst + kr + ke), Tp: kst, T0: kst, Tm: kst}
    off[Tp] = {Tp: -(2 * kst + ke), S: kst, T0: kst}
    off[T0] = {T0: -(3 * kst + ke), S: kst, Tp: kst, Tm: kst}
    off[Tm] = {Tm: -(2 * kst + ke), S: kst, T0: kst}
```

```
on = {}
on[S] = {S: -(kst + 2 * krlx + kr + ke), Tp: krlx, T0: kst, Tm: krlx}
on[Tp] = {Tp: -(2 * krlx + ke), S: krlx, T0: krlx}
on[T0] = {T0: -(kst + 2 * krlx + ke), S: kst, Tp: krlx, Tm: krlx}
on[Tm] = {Tm: -(2 * krlx + ke), S: krlx, T0: krlx}

initial_states = {Tp: 1 / 3, T0: 1 / 3, Tm: 1 / 3}
time = np.linspace(0, 1e-6, 10000)

roff = RateEquations(off)
ron = RateEquations(on)
result_off = RateEquations.time_evolution(roff, time, initial_states)
result_on = RateEquations.time_evolution(ron, time, initial_states)

keys = [S, Tp, T0, Tm]
rp_field_off = result_off[keys]
rp_field_on = result_on[keys]
rp_delta_delta_A = rp_field_on - rp_field_off

plt.clf()
fig = plt.figure()
scale = 1e6
gs = fig.add_gridspec(2, hspace=0)
axs = gs.subplots(sharex=True)
fig.suptitle("Triplet born radical pair", size=18)
axs[0].plot(time * scale, rp_field_off, color="blue", linewidth=2)
axs[0].plot(time * scale, rp_field_on, color="green", linewidth=2)
axs[1].plot(time * scale, rp_delta_delta_A, color="orange", linewidth=2)
plt.xscale("linear")
axs[0].legend([r"$F (B_0 = 0)$", r"$F (B_0 \neq 0)$"])
axs[1].set_xlabel("Time ($\mu s$)", size=14)
axs[0].set_ylabel("$\Delta A$", size=14)
axs[1].set_ylabel("$\Delta \Delta A$", size=14)
```

```
    axs[0].tick_params(labelsize=14)
    axs[1].tick_params(labelsize=14)
    fig.set_size_inches(10, 5)
    path = __file__[:-3] + f"_{0}.png"
    plt.savefig(path)

    print(latex_eqlist_to_align(latexify(off)))
    print(latex_eqlist_to_align(latexify(on)))


if __name__ == "__main__":
    main()
```

Figure S2

```python
#! /usr/bin/env python


import matplotlib.pyplot as plt
import numpy as np
import tqdm
import radicalpy as rp



def main():
    # Monte Carlo random walk simulation.

    # parameters
    np.random.seed(42)
    t = np.arange(0, 40e-9, 40e-12)
    r_min = 5e-10 / 2
    r_max = 10e-10
    r = (r_min) + np.random.sample() * ((r_max) - (r_min))
    x0, y0, z0 = r, 0, 0
    mutual_diffusion = 1e-6 / 10000
    N = 600

    delta_r = rp.classical.get_delta_r(mutual_diffusion, t[1] - t[0])
    dist, ang = np.zeros([N, len(t)]), np.zeros([N, len(t)])

    for i in tqdm.tqdm(range(0, N)):
        pos, dist[i], ang[i] = rp.classical.randomwalk_3d(
            len(t), x0, y0, z0, delta_r, r_min, r_max
        )

    rp.plot.monte_carlo_caged(pos, r_max)
    path = __file__[:-3] + f"_{0}.png"
```

```
plt.savefig(path)

t_convert = 1e-9
point = 4
dist_av = np.ndarray.mean(dist, axis=0)

# plt.set_facecolor("none")
plt.clf()
plt.grid(False)
plt.axis("on")
plt.rc("axes", edgecolor="k")
plt.plot(t[point:] / t_convert, dist_av[point:] / 1e-9, "r")
plt.title("Time evolution of radical pair separation", size=16)
plt.xlabel("$t$ ($ns$)", size=14)
plt.ylabel("$r$ (nm)", size=14)
plt.tick_params(labelsize=14)
path = __file__[:-3] + f"_{1}.png"
plt.savefig(path)

J_all = rp.estimations.exchange_interaction_in_solution_MC(dist)
J_av = np.ndarray.mean(J_all, axis=0)

# plt.set_facecolor("none")
plt.clf()
plt.grid(False)
plt.axis("on")
plt.rc("axes", edgecolor="k")
plt.plot(t[point:] / t_convert, J_av[point:])
plt.title("Time evolution of the exchange interaction", size=16)
plt.xlabel("$t$ ($ns$)", size=14)
plt.ylabel("$J$ (mT)", size=14)
plt.tick_params(labelsize=14)
path = __file__[:-3] + f"_{2}.png"
```

```
    plt.savefig(path)

    D_all = rp.estimations.dipolar_interaction_MC(dist, ang)
    D_av = np.ndarray.mean(D_all, axis=0)

    # plt.facecolor("none")
    plt.clf()
    plt.grid(False)
    plt.axis("on")
    plt.rc("axes", edgecolor="k")
    plt.plot(t[point:] / t_convert, D_av[point:], "g")
    plt.title("Time evolution of the dipolar interaction", size=16)
    plt.xlabel("$t$ ($ns$)", size=14)
    plt.ylabel("$D$ (mT)", size=14)
    plt.tick_params(labelsize=14)
    path = __file__[:-3] + f"_{3}.png"
    plt.savefig(path)


if __name__ == "__main__":
    main()
```

References

(1) Hayashi, H.; Nagakura, S. Theoretical Study of Relaxation Mechanism in Magnetic Field Effects on Chemical Reactions. *Bull. Chem. Soc. Jpn.* **1984**, *57*, 322–328.

(2) Steiner, U. E.; Ulrich, T. Magnetic field effects in chemical kinetics and related phenomena. *Chem. Rev.* **1989**, *89*, 51–147.

(3) Turro, N. J. Micelles, magnets and molecular mechanisms. Application to cage effects and isotope separation. *J. Macromol. Sci. Part A Pure Appl. Chem.* **1981**, *53*, 259–286.

(4) Gould, I. R.; Zimmt, M. B.; Turro, N. J.; Baretz, B. H.; Lehr, G. F. Dynamics of radical pair reactions in micelles. *J. Am. Chem. Soc.* **1985**, *107*, 4607–4612.

(5) Antill, L. M.; Takizawa, S.-Y.; Murata, S.; Woodward, J. R. Photoinduced flavin-tryptophan electron transfer across vesicle membranes generates magnetic field sensitive radical pairs. *Mol. Phys.* **2018**, *117*, 2594–2603.

(6) Yago, T.; Ishii, Y.; Wakasa, M. Diffusion and Solvation of Radical Ions in an Ionic Liquid Studied by the MFE Probe. *J. Phys. Chem. C* **2014**, *118*, 22356–22367.

(7) Maeda, K.; Robinson, A. J.; Henbest, K. B.; Hogben, H. J.; Biskup, T.; Ahmad, M.; Schleicher, E.; Weber, S.; Timmel, C. R.; Hore, P. J. Magnetically sensitive light-induced reactions in cryptochrome are consistent with its proposed role as a magnetoreceptor. *Proc. Natl. Acad. Sci. USA* **2012**, *109*, 4774–4779.

(8) O'Dea, A. R.; Curtis, A. F.; Green, N. J. B.; Timmel, C. R.; Hore, P. J. Influence of dipolar interactions on radical pair recombination reactions subject to weak magnetic fields. *J. Phys. Chem. A* **2005**, *109*, 869–873.

(9) Miura, T.; Murai, H. Effect of molecular diffusion on the spin dynamics of a micellized radical pair in low magnetic fields studied by Monte Carlo simulation. *J. Phys. Chem. A* **2015**, *119*, 5534–5544.

(10) Kattnig, D. R.; Sowa, J. K.; Solov'yov, I. A.; Hore, P. J. Electron spin relaxation can enhance the performance of a cryptochrome-based magnetic compass sensor. *New J. Phys.* **2016**, *18*, 063007.

(11) Shushin, A. I. The effect of the spin exchange interaction on SNP and RYDMR spectra of geminate radical pairs. *Chem. Phys. Lett.* **1991**, *181*, 274–278.

(12) Hogben, H. J.; Krzystyniak, M.; Charnock, G. T. P.; Hore, P. J.; Kuprov, I. Spinach–a software library for simulation of spin dynamics in large spin systems. *J. Magn. Reson.* **2011**, *208*, 179–194.

(13) Frisch, M. J.; Trucks, G. W.; Schlegel, H. B.; Scuseria, G. E.; Robb, M. A.; Cheeseman, J. R.; Scalmani, G.; Barone, V.; Petersson, G. A.; Nakatsuji, H.; Li, X.; Caricato, M.; Marenich, A. V.; Bloino, J.; Janesko, B. G.; Gomperts, R.; Mennucci, B.; Hratchian, H. P.; Ortiz, J. V.; Izmaylov, A. F.; Sonnenberg, J. L.; Williams-Young, D.; Ding, F.; Lipparini, F.; Egidi, F.; Goings, J.; Peng, B.; Petrone, A.; Henderson, T.; Ranasinghe, D.; Zakrzewski, V. G.; Gao, J.; Rega, N.; Zheng, G.; Liang, W.; Hada, M.; Ehara, M.; Toyota, K.; Fukuda, R.; Hasegawa, J.; Ishida, M.; Nakajima, T.; Honda, Y.; Kitao, O.; Nakai, H.; Vreven, T.; Throssell, K.; Montgomery, J. A., Jr.; Peralta, J. E.; Ogliaro, F.; Bearpark, M. J.; Heyd, J. J.; Brothers, E. N.; Kudin, K. N.; Staroverov, V. N.; Keith, T. A.; Kobayashi, R.; Normand, J.; Raghavachari, K.; Rendell, A. P.; Burant, J. C.; Iyengar, S. S.; Tomasi, J.; Cossi, M.; Millam, J. M.; Klene, M.; Adamo, C.; Cammi, R.; Ochterski, J. W.; Martin, R. L.; Morokuma, K.; Farkas, O.; Foresman, J. B.; Fox, D. J. Gaussian 16 Revision C.01. 2016; Gaussian Inc. Wallingford CT.

(14) Neese, F.; Wennmohs, F.; Becker, U.; Riplinger, C. The ORCA quantum chemistry program package. *J. Chem. Phys.* **2020**, *152*, 224108.

(15) Santabarbara, S.; Kuprov, I.; Fairclough, W. V.; Purton, S.; Hore, P. J.; Heathcote, P.;

Evans, M. C. W. Bidirectional electron transfer in photosystem I: determination of two distances between P700+ and A1- in spin-correlated radical pairs. *Biochemistry* **2005**, *44*, 2119–2128.

(16) Déjean, V.; Konowalczyk, M.; Gravell, J.; Golesworthy, M. J.; Gunn, C.; Pompe, N.; Foster Vander Elst, O.; Tan, K.-J.; Oxborrow, M.; Aarts, D. G. A. L.; Mackenzie, S. R.; Timmel, C. R. Detection of magnetic field effects by confocal microscopy. *Chem. Sci.* **2020**, *11*, 7772–7781.

(17) Efimova, O.; Hore, P. J. Role of exchange and dipolar interactions in the radical pair model of the avian magnetic compass. *Biophys. J.* **2008**, *94*, 1565–1574.

(18) Kattnig, D. R.; Hore, P. J. The sensitivity of a radical pair compass magnetoreceptor can be significantly amplified by radical scavengers. *Sci. Rep.* **2017**, *7*, 11640.

(19) Haberkorn, R. Density matrix description of spin-selective radical pair reactions. *Mol. Phys.* **1976**, *32*, 1491–1493.

(20) Jones, J. A.; Maeda, K.; Hore, P. J. Reaction operators for spin-selective chemical reactions of radical pairs. *Chem. Phys. Lett.* **2011**,

(21) Gorelik, V. R.; Maeda, K.; Yashiro, H.; Murai, H. Microwave-Induced Quantum Beats in Micellized Radical Pairs under Spin-Locking Conditions. *J. Phys. Chem. A* **2001**, *105*, 8011–8017.

(22) Bloch, F. Nuclear Induction. *Phys. Rev.* **1946**, *70*, 460–474.

(23) Kivelson, D. Theory of ESR Linewidths of Free Radicals. *J. Chem. Phys.* **1960**, *33*, 1094–1106.

(24) Brenes, M.; Varma, V. K.; Scardicchio, A.; Girotto, I. Massively Parallel Implementation and Approaches to Simulate Quantum Dynamics Using Krylov Subspace Techniques. *Comput. Phys. Commun.* **2019**, *235*, 477–488.

(25) Schulten, K.; Wolynes, P. G. Semiclassical description of electron spin motion in radicals including the effect of electron hopping. *J. Chem. Phys.* **1978**, *68*, 3292–3297.

(26) Manolopoulos, D. E.; Hore, P. J. An improved semiclassical theory of radical pair recombination reactions. *J. Chem. Phys.* **2013**, *139*, 124106.

(27) Fay, T. P.; Lindoy, L. P.; Manolopoulos, D. E.; Hore, P. J. How quantum is radical pair magnetoreception? *Faraday Discuss.* **2019**, *221*, 77–91.

(28) Islam, S. D. M.; Susdorf, T.; Penzkofer, A.; Hegemann, P. Fluorescence quenching of flavin adenine dinucleotide in aqueous solution by pH dependent isomerisation and photo-induced electron transfer. *Chem. Phys.* **2003**, *295*, 137–149.

(29) Murakami, M.; Maeda, K.; Arai, T. Dynamics of intramolecular electron transfer reaction of FAD studied by magnetic field effects on transient absorption spectra. *J. Phys. Chem. A* **2005**, *109*, 5793–5800.

(30) Antill, L. M.; Woodward, J. R. Flavin Adenine Dinucleotide Photochemistry Is Magnetic Field Sensitive at Physiological pH. *J. Phys. Chem. Lett.* **2018**, *9*, 2691–2696.

(31) Maeda, K.; Miura, T.; Arai, T. A practical simulation and a novel insight to the magnetic field effect on a radical pair in a micelle. *Mol. Phys.* **2006**, *104*, 1779–1788.
